# Exploring patient and provider perspectives on outpatient care and the family practice model at a university hospital in Ghana

**DOI:** 10.1371/journal.pone.0327277

**Published:** 2026-09-09

**Authors:** Abigail Kusi-Amponsah Diji, Nana Kwame Ayisi-Boateng, Hayford Asare, Godwin Antwi, Douglas Aninng Opoku, Eric Kojo Nsa Oduro, Emmanuel Konadu, Nana Akua Abruquah, Elizabeth Oppong-Kyekyeku, Mercy Addae, Emmanuel Mfum-Mensah, Anita Asiwome Baku

**Affiliations:** 1 School of Nursing and Midwifery, Kwame Nkrumah University of Science and Technology, Kumasi, Ghana; 2 University Hospital, Kwame Nkrumah University of Science and Technology, Kumasi, Ghana; 3 School of Medical Sciences, Kwame Nkrumah University of Science and Technology, Kumasi, Ghana; 4 School of Public Health, Kwame Nkrumah University of Science and Technology, Kumasi, Ghana; 5 University Information and Technological Services, Kwame Nkrumah University of Science and Technology, Kumasi, Ghana; 6 University of Ghana Business School, University of Ghana, Legon, Ghana; North-West University, SOUTH AFRICA

## Abstract

**Background:**

Fragmented outpatient care, and disrupted patient–provider continuity hinder progress towards the achievement of Universal Health Coverage (UHC) in many low- and middle-income countries including Ghana. Despite efforts such as the National Health Insurance Scheme and Community-based Health Planning and Services, continuity of care remains a challenge. This study explored stakeholders’ perspectives on implementing a Family Practice Model to strengthen primary care and advance UHC at a University Hospital in Ghana.

**Methods:**

A descriptive-analytical qualitative study was conducted from September 2023 to May 2024 at the University Hospital, Kwame Nkrumah University of Science and Technology, Kumasi. Twenty-eight participants were purposively selected, comprising nine healthcare providers (physicians and physician assistants) and 19 care recipients (university staffs). Semi-structured interviews, guided by the Consolidated Framework for Implementation Research, explored experiences with the current outpatient system and views on the proposed FPM. Data were audio-recorded, transcribed verbatim, and thematically analyzed using NVivo version 14.

**Results:**

Two major themes emerged: “Therapeutic Disconnection” and “A Doctor to Call My Own.” Participants described the existing care system as fragmented, impersonal, and marked by rotating providers, poor documentation, and rushed consultations. These issues weakened trust and care continuity. In contrast, stakeholders supported a dedicated, relationship-based model that would ensure continuity, streamline care processes, and foster long-term provider–patient connections.

**Conclusion:**

The Family Practice Model could transform outpatient care at the university hospital and beyond by enhancing continuity, trust, and overall service quality. However, successful implementation depends on tailored strategies encompassing policy support, and pilot testing to assess feasibility and impact on UHC goals in Ghana.

## Introduction

Primary healthcare is widely recognized as the cornerstone of effective health systems, as it enhances essential services, strengthens disease prevention, and ensures coordinated care throughout a person’s life course [1–3]. The outpatient department (OPD) often serves as the critical first point of contact for non-emergency medical issues. Nevertheless, the structure and quality of OPD services vary considerably across countries due to differences in resource allocation, provider training, and organizational models, particularly in low- and middle-income countries (LMICs) such as Ghana [4]. In many Ghanaian healthcare settings, OPD services operate on a fragmented, episodic model where patients see different providers at each visit. This often leads to repeated recounting of medical histories and undermines the development of trust and continuity [5,6]. This fragmentation has prompted calls for innovative healthcare delivery models that emphasize long-term relationships, seamless coordination, and comprehensive care [7].

One promising approach is the Family Practice Model (FPM), in which a designated physician provides continuous, comprehensive, and personalized care to an individual and their family [8,9]. In contrast to episodic care in the conventional OPD setting, the FPM establishes continuity by enabling regular monitoring of medical histories, family health patterns, and psychosocial factors, which are essential for effective chronic disease management [10]. Evidence from high-income countries such as Canada, and the United Kingdom indicates that such a model not only improves patient satisfaction and clinical outcomes but also reduces hospitalizations and overall healthcare costs [11,12]. By proactively coordinating care to match individual and family health needs, the FPM aligns with best practices for chronic disease management and prevention [9,10].

In Ghana and Sub-Saharan Africa, many OPD systems are constrained by generic, one-off consultations [13,14]. This care structure disrupts continuity by compelling patients to repeatedly recount their medical and social histories, thereby diminishing trust in healthcare providers [15]. For healthcare providers, this fragmented system intensifies workload demands and increases the risk of burnout, ultimately hindering their ability to deliver relationship-centered care that is critical for high-quality service provision [5,6]. These challenges are particularly pronounced in hospitals, where high patient volumes and complex cases demand more coordinated, personalized approaches to care.

In response to these challenges, the University Hospital of the Kwame Nkrumah University of Science and Technology (KNUST) in Ghana is exploring the adoption of a FPM in its OPD. This model of out-patient care is intended to remedy current shortcomings by enhancing continuity of care, offering personalized services, and providing comprehensive management of chronic conditions [8]. However, achieving a successful transition necessitates a thorough understanding of stakeholders’ perspectives. It is therefore essential to gather insights from healthcare providers and key recipients of care (university staff and their dependents) regarding (1) their satisfaction with the already established OPD system, (2) their views on the feasibility and anticipated benefits of the Family Practice Model and (3) the potential challenges that may arise during its implementation to inform policy decisions and guide the piloting or scaling of the FPM.

## Materials and methods

### Theoretical model

The Consolidated Framework for Implementation Research (CFIR), developed by Damschroder et al. [16] served as the theoretical foundation for both the design and analysis phases of this study. CFIR offers a comprehensive and systematic structure for identifying factors that influence the successful implementation of complex healthcare interventions. It is organized around five interrelated domains: Intervention Characteristics, Outer Setting, Inner Setting, Characteristics of Individuals, and Process. Each domain includes a range of constructs that enable researchers to assess contextual, organizational, and individual-level influences that may act as facilitators or barriers to implementation.

In this study, which explored stakeholder perceptions of introducing the FPM within the OPD of KNUST Hospital, CFIR provided a structured lens for assessing the acceptability, feasibility, and contextual appropriateness of the proposed model. For example, the Intervention Characteristics domain guided the examination of stakeholder perceptions regarding the FPM’s relative advantage, complexity, and adaptability compared to the OPD system that had been in place in the hospital for years. The Outer Setting domain allowed for the exploration of external factors such as patient needs, community expectations, and broader healthcare policy influences. The Inner Setting focused on the hospital’s organizational culture, communication patterns, resource availability, and readiness for change. The Characteristics of Individuals domain captured the knowledge, beliefs, and attitudes of healthcare providers and recipients that might shape their engagement with the FPM. Finally, the Process domain offered insights into potential implementation strategies, including planning, stakeholder involvement, and mechanisms for ongoing evaluation.

CFIR also informed the development of the semi-structured interview guides, ensuring that questions were aligned with the framework’s key domains. This facilitated a comprehensive exploration of stakeholder perspectives, including perceived benefits, challenges, and practical considerations for implementation. During data analysis, CFIR served as a deductive coding framework, enabling the categorization of responses into predefined domains. At the same time, an inductive approach was applied to capture emergent themes that were not anticipated during the design phase. This hybrid analytical strategy ensured that the findings were both theoretically grounded and reflective of the real-world context of healthcare delivery in a university hospital setting.

### Study design

We employed a descriptive-analytical qualitative design to explore stakeholder perspectives on implementing the FPM in a University Hospital. This approach was selected to generate rich, contextual insights into a phenomenon with limited prior investigation [17,18], particularly regarding healthcare provider and recipient’s perspectives on the current OPD and the proposed FPM. A qualitative design was most appropriate given the study’s focus on understanding experiences, perceptions, and contextual factors shaping healthcare delivery, rather than producing generalizable estimates. This design thus provided an in-depth, exploratory foundation to inform future mixed-methods or large-scale studies evaluating the FPM’s feasibility and impact.

### Study setting

The study was conducted at the University Hospital, KNUST in Kumasi, Ghana. The University Hospital is a 135-bed district-level facility that provides healthcare services to a diverse population, including members of the university community (students, faculty, and staff) as well as residents of surrounding neighbourhoods such as Ayigya, Bomso, Ayeduase, and Kentinkrono. The hospital offers a wide range of outpatient and inpatient services and serves as both a healthcare delivery centre and a clinical training site for health sciences students. As a primary care facility offering general and specialized services (such as gynaecology, obstetrics, paediatrics, surgery, emergency, etc.), the hospital employs over 300 staffs including multidisciplinary health professionals [19].

This setting was considered ideal due to its dual role as a teaching and service-oriented hospital, serving a diverse and stable patient population. Additionally, the hospital reflects broader systemic challenges in Ghana’s outpatient care, including fragmented and impersonal services. Its strong institutional commitment to innovation, emphasis on strengthening primary healthcare, and accessibility for research further reinforced its suitability as an ideal site for this study.

### Participant recruitment

A purposive sampling strategy with a maximum variation technique was employed to ensure a broad representation of participants across key characteristics such as college or departmental affiliation, staff category (academic and non-academic), professional rank, and gender. This approach was intended to capture diverse perspectives on the current OPD system and the proposed FPM.

Two main stakeholder groups were targeted:

- Healthcare Providers: This group included medical doctors and physician assistants with a minimum of one year of professional experience at KNUST. This criterion ensured that participants had adequate familiarity with existing OPD processes and could provide informed perspectives on the feasibility and implications of introducing the FPM.
- Healthcare Recipients: This group comprised university staff, including senior and junior members, who had accessed OPD services at KNUST Hospital within the past year. Their recent experiences positioned them to offer meaningful insights into their satisfaction and expectations for improved continuity of care. The inclusion of only university staff was deliberate, as they constitute the hospital’s core and most consistent outpatient population, whose experiences reflect the dominant service patterns and challenges within the facility. Additionally, focusing on this group provided a controlled and homogeneous context for exploring the feasibility of introducing the FPM before extending it to the general surrounding communities served by the hospital.

Potential participants were initially identified through hospital records, after which they were sent text messages containing a brief description of the study and a link through which they could confirm their interest in participating. Those who agreed to take part were then contacted by the research team to schedule a private, in-person interview at a convenient time and location. This recruitment process facilitated the inclusion of information-rich participants with relevant experience, while also respecting participants’ autonomy and availability, ultimately contributing to the rigour and depth of the qualitative inquiry.

The final sample included nine healthcare providers and nineteen healthcare recipients. The sample size was guided by the principle of data saturation, which was reached after the 8th healthcare provider and 17th healthcare recipient interview when no new information or themes emerged from additional interviews. This number is consistent with qualitative research conventions recommending 15–30 participants to achieve thematic saturation in exploratory studies of this nature [20]. Thus, the sample was considered sufficient to ensure credibility, diversity, and transferability of findings rather than statistical generalizability.

### Data collection and analysis

Between September 2023 and May 2024, individual face-to-face interviews were conducted with healthcare providers and recipients (university staff) in English or Twi (the predominantly spoken local language), depending on the participant’s preference. Interviews conducted in Twi were transcribed and translated into English by bilingual members of the research team who were fluent in both languages. The accuracy of the translations was validated by a Twi language expert, and all transcripts were cross-checked against the original audio recordings to ensure fidelity and accuracy. The semi-structured interviews were guided by an interview protocol developed through a comprehensive review of relevant literature [21]. The guide was aligned with the study’s objectives and focused on two main domains namely: (1) experiences with the existing OPD system, and (2) acceptability, feasibility, and contextual appropriateness of the FPM (refer to S1 Appendix).

Two tailored versions of the guide were created: one for healthcare providers (doctors and physician assistants), and another for the recipients of care (university staff). Each version included a brief socio-demographic section followed by open-ended questions with prompts to explore experiences with the existing OPD system, perceptions of the proposed FPM, and recommendations for improvement. The approach allowed interviewers to probe further based on participants’ responses, ensuring both consistency and depth across interviews. The interview guide was pre-tested among 14 participants, comprising seven healthcare providers and seven healthcare recipients from a similar hospital setting. Their feedback was used to improve the clarity, sequencing, relevance and flow of the questions as the overall focus of the guide remained unchanged. Three trained research assistants (two males and one female) with expertise in qualitative interviews facilitated the sessions, which lasted for 30–45 minutes in quiet, private areas and offices. Prior to beginning each interview, brief demographic information from participants was collected to contextualize their responses. The interview sessions were audio-recorded, and comprehensive field notes were taken concurrently to capture non-verbal cues and contextual details that could enrich the data analysis. All the audio recordings were transcribed verbatim and anonymized; the transcripts were subsequently stored securely on an encrypted drive accessible only to the research team. Hard copies of the research data were kept in locked storage facilities within the Principal Investigator’s office.

Data collection and analysis were carried out concurrently, allowing for iterative refinement of interview questions and active generation of codes, sub-themes/categories and themes. They continued until no new patterns or insights emerged from subsequent interviews, indicating that thematic saturation had been achieved. This ensured that the dataset captured sufficient depth and variation to address the study objectives. A hybrid approach that combines both inductive and deductive thematic analysis guided by the CFIR was used to balance structured exploration with an existing framework and the flexibility to allow for the active generation of themes from participants’ narratives. This approach was adopted to ensure that CFIR provided a structured deductive lens for examining implementation-related domains, while the inductive component allowed context-specific insights to emerge beyond the framework. This was particularly important given the limited evidence on the Family Practice Model in this setting, where relying solely on predetermined CFIR constructs could have overlooked locally relevant experiences, perceptions, and implementation challenges. The inductive thematic analysis process followed the six-phase framework proposed by Braun and Clarke [22]: (1) familiarization with the data, (2) generation of initial codes, (3) searching for themes, (4) reviewing themes, (5) defining and naming themes, and (6) producing the report. Codes generated inductively were later compared with CFIR-informed categories to identify areas of overlap or divergence, enabling a more holistic understanding of stakeholders’ perspectives. The qualitative data analysis and organization were facilitated with NVivo version 12 software. Data saturation for both groups (healthcare providers and recipients) were confirmed when participants consistently expressed similar experiences and views regarding the phenomenon under study, with no new perspectives emerging during the analysis of later interviews.

### Rigour

To enhance the study’s methodological rigour, Guba and Lincoln’s principles of credibility, dependability, confirmability, and transferability were adhered to throughout the study period [23]. Credibility was achieved through prolonged engagement with participants, and member-checking was conducted for each transcript to ensure that participants’ perspectives were accurately represented and interpreted [24,25]. This iterative validation process was consistent with established qualitative research standards that emphasize sustained interaction with data and participants [26].

Dependability was ensured by keeping a detailed audit trail that systematically recorded all steps taken during the data analysis process. This involved keeping all materials and documentation related to the analysis process such as the original audio recordings of interviews, written transcripts, observational field notes, and detailed logs showing how codes, sub-themes/categories and themes were developed, revised, or refined throughout the analysis. These detailed records allowed for peer debriefing, during which research decisions and interpretations were critically examined by the research team, enhancing the transparency and consistency of the analytical process over time [27,28].

Confirmability was enhanced using dual coding, whereby two independent researchers (GA and HA) coded the data separately. This approach allowed for a comparison of interpretations, reducing the influence of individual bias and increasing the trustworthiness of the findings. By cross-checking and discussing their analyses, the researchers ensured that the identified themes were clearly supported by the raw data [29]. This strategy aligns with best practices in qualitative research, which emphasize inter-coder agreement as a means of strengthening the accuracy and objectivity of data interpretation.

Transferability was achieved by providing thick, detailed description of the study’s context, the characteristics of the participants, and the data collection procedures. This rich contextual information allows readers to assess the applicability of the findings to similar settings, thereby enhancing the study’s external validity [30].

Transferability was addressed by providing thick, detailed description of the study’s context, including detailed accounts of the research setting, the demographic and professional characteristics of the participants, and the specific procedures used during data collection. This rich contextual information provides readers with sufficient information to determine the applicability of the findings to similar settings, thereby enhancing the study’s external validity [30].

### Ethical considerations

This study received ethical approval from the Committee on Human Research, Publications and Ethics (CHRPE) of KNUST (Reference Number: CHRPE/AP/716/23) as well as institutional permission from the University Hospital management. The study adhered to ethical principles of informed consent, voluntary participation, confidentiality, secure data handling, and anonymized reporting, thereby ensuring that participants’ dignity and privacy were upheld throughout the research process.

Participation in the study was voluntary, and informed consent was obtained in writing from each participant prior to data collection. A detailed information sheet explaining the study’s purpose, data collection procedures, rights of participants, potential risks and benefits was provided to all participants. Confidentiality, anonymity and respect for participant’s privacy were also ensured.

## Results

### Sociodemographic characteristics of healthcare providers

A total of nine healthcare providers, comprising of six medical officers and three physician assistants. Their ages ranged from 26 to 46 years (refer to Table 1). Most of the participants were males (n = 7,77.8%), with more than half of them being married (n = 5,55.6%). While four of the participating healthcare providers had no child, five of them had between two to three children. All of them had worked for one to three years in their respective units at the Hospital.

**Table 1:** Sociodemographic characteristics of healthcare providers.

Participant	Age (years)	Gender	Marital status	Rank	Number of children	Unit	Working years the Unit
PA1	26-30	Female	Not married	Physician Assistant	None	KNUST Hospital	3
PA2	26-30	Male	Not married	Physician Assistant	None	KNUST Hospital	3
PA3	31-35	Female	Not married	Physician Assistant	None	KNUST Hospital	1
MO1	31-35	Male	Not married	Medical officer	None	Emergency department	1
MO2	36-40	Male	Married	Senior Medical Officer	None	Family medicine	1
MO3	36-40	Male	Married	Medical Officer	3	KNUST Hospital	3
MO4	31-35	Male	Married	Medical Officer	2	KNUST Hospital	3
MO5	46-50	Male	Married	Medical Officer	3	KNUST Hospital	2
MO6	31-35	Male	Married	Medical Officer	3	KNUST Hospital	3

### Sociodemographic characteristics of healthcare recipients

A total of 19 healthcare recipients, consisting of six senior members, seven senior staff, and six junior members. Their ages ranged from 30 and 65 years, majority of them being males (n = 10, 52.6%). Most of the participants were married with at least one child (n = 16,84.2%). Participants were from diverse colleges and units including Engineering (n = 2,10.5%), Agricultural and Natural Resources (n = 2,10.5%), Humanities and Social Sciences (n = 2,10.5%), Health Sciences (n = 2,10.5%), Science (n = 2,10.5%), Arts and Built Environment (n = 1, 5.3%), Security Unit (n = 2, 10.5%), and Cleaning Unit (n = 4,21.1%). At the time of the study, they had been receiving healthcare services for a minimum period of one year to a maximum period of 40 years (refer to Table 2).

**Table 2:** Sociodemographic characteristics of senior members, senior staff, and junior members.

Participant	Age (years)	Gender	Marital status	Number of children	College/Faculty	Working Years in Healthcare
	**Senior members**					
SM1	41-45	Male	Married	3	Humanities and Social Science	3
SM2	31-35	Female	Married	2	Engineering	3
SM3	36-40	Male	Married	4	Science	2
SM4	51-55	Male	Married	4	Agricultural and Natural Resources	13
SM5	61-65	Male	Married	5	Art and Built Environment	32
SM6	36-40	Male	Married	4	Health Science	13
	**Senior staffs**					
SS1	46-50	Female	Married	3	Arts and Built Environment	>20
SS2	41-45	Female	Married	3	Engineering	10
SS3	56-60	Female	Married	6	Faculty of Education	40
SS4	46-50	Female	Married	2	Science	1
SS5	31-35	Male	Married	1	Health sciences	3
SS6	31-35	Female	Not married	None	Humanities and Social Sciences	10
SS7	56-60	Male	Married	6	Agriculture and Natural Resources	23
	**Junior staffs**					
JS1	41-45	Male	Married	5	Security	8
JS2	46-50	Female	Married	5	Cleaning services	25
JS3	51-55	Female	Not married	2	Security	16
JS4	41-45	Female	Married	3	Cleaning services	15
JS5	56-60	Male	Married	2	Cleaning services	8
JS6	26-30	Male	Not married	None	Cleaning services	1

### Themes

Two themes were actively generated from the data namely: “Therapeutic disconnection” and “A doctor to call my own” (refer to Table 3). These themes captured the essence of healthcare experiences regarding the existing OPD system and aspirations expressed by both patients and providers on the proposed new FPM. Using the Consolidated Framework for Implementation Research (CFIR) as a guiding lens, the findings highlight how contextual factors within the Inner Setting, Outer Setting, Process, and Intervention Characteristics domains influence the feasibility and potential success of the FPM.

**Table 3:** Themes and sub-themes from healthcare providers and healthcare recipients.

Themes	Sub-themes
Therapeutic disconnection	Care on the clock
	Fragmented care
	Care without comfort
A doctor to call my own	Healing through continuity
	Beneath the optimism
	Operationalizing the vision

#### Theme 1: Therapeutic disconnection.

This theme described the shared experiences of both healthcare providers and recipients who recounted a profound lack of continuity, emotional connection, and personalized care within the existing outpatient department (OPD) system. This theme captured how rotating physicians and physician assistants, missing records, and rushed encounters led to fragmented clinical relationships and weakened trust in the care process. Healthcare recipient participants revealed that, despite being “seen,” they often felt unseen, unknown, and unremembered during their hospital encounters. Three sub-themes were generated from this theme namely: “care on the clock”, “fragmented care”, and “care without comfort” (Refer to Table 3). These findings are directly linked to the Consolidated Framework for Implementation Research (CFIR), particularly the Inner Setting domain, which reflects how organizational structures, workflow patterns, communication systems, and continuity of care processes within the hospital contributed to the observed therapeutic disconnection. In addition, elements of the Outer Setting are also evident, particularly in relation to patient expectations for continuous, respectful, and person-centred care, shaped by broader societal norms and experiences with healthcare delivery.

### Care on the clock

A third and significant dimension of therapeutic disconnection, as recounted by participants, was the experience of receiving or delivering time-constrained, high-paced medical care that left little room for relational engagement or critical reflection. This sub-theme, “*Care on the Clock*”, captured how the structural design of the OPD system which is built around efficiency, volume, and speed, contributed to a sense of clinical fragmentation, even before emotional disconnection occurred. This sub-theme is directly linked to the CFIR Inner Setting domain, particularly structural characteristics, workflow constraints, and resource limitations that shape how care is delivered within the OPD.

Healthcare providers repeatedly described the pace of their work as unsustainable and dehumanizing, particularly during early morning clinics, after weekends, or during high-traffic periods such as end-of-month or payroll weeks. One medical officer working in the General OPD recounted that she often attended to more than 50 patients per shift, sometimes without taking a single break. She explained,

*“You don’t have time to breathe. You just keep going. And after a point, you’re not thinking … you’re just surviving (M01,healthcare provider).”*

Her words conveyed not just the pressure of time, but the cognitive fatigue and emotional detachment that accompanied such pace.

From the recipient’s perspective, the same dynamics manifested as rushed consultations and shallow interactions. Several patients described spending two to three hours in queues, only to be seen for less than five minutes. A senior member from the College of Science shared this:

*“I would get there at 7 a.m., and sometimes I was seen at 10. But the actual consultation would be two minutes. It felt like I waited all morning just to be pushed out (SM3, healthcare recipient).”*

This sentiment of being “processed” rather than cared for was echoed across multiple narratives. The pressure of time also affected the content of consultations. A provider in the Surgical Unit mentioned that, due to the speed required, they often focused narrowly on the presenting complaint, even when it was clear that the patient had underlying concerns. He recounted:

*“Sometimes I know there’s more going on … but I just don’t have the time to ask. (MO6, healthcare provider)”*

For patients, this meant that care often addressed symptoms but not stories, and they left the hospital with questions unanswered or concerns unspoken.

### Fragmented care

The sub-theme, “Fragmented Care”, was characterized by a structural reality where clinical and therapeutic continuity was undermined by inconsistent physician assignment, rotating staff schedules, and gaps in documentation. This reflects the CFIR Inner Setting domain, particularly communication networks, workflow processes, and information systems that shape continuity of care within the OPD. It also reflects, to a lesser extent, Outer Setting expectations where patients anticipate continuity and coordinated care as part of acceptable service delivery, highlighting a mismatch between expectations and actual service experience. This was a dominant thread running through the narratives of both healthcare providers and recipients, particularly within the outpatient department (OPD) setting. Participants consistently described a healthcare system in which *continuity of care was the exception, not the norm (SM1, healthcare recipient; MO1, healthcare provider)”*. Rather than encountering a familiar provider with each visit, recipients of care often found themselves retelling their stories to new doctors, while healthcare professionals expressed frustration at being unable to follow up with patients they had previously treated.

Recipients of care frequently recounted how their experiences with the OPD felt disconnected and repetitive. Many described the emotional toll of constantly re-introducing themselves, rehashing their symptoms, or re-explaining their treatment history to a succession of unfamiliar doctors. As one recipient from the College of Agriculture shared,

*“Each time I went, it was someone new. I had to explain everything all over again. It was exhausting. (SM4, healthcare recipient)”*

This repetitive cycle, for many patients, not only delayed care but also eroded their trust in the system. A senior academic from the College of Humanities reflected on how this inconsistency affected her chronic condition:

*“I had a doctor who knew my case very well, but when she went on leave, I had to start again with someone else. There was no transition (SM1, healthcare recipient).”*

On the providers’ side, similar challenges were voiced. Medical officers across departments, particularly in General OPD, Surgery, and Family Medicine, described clinical inefficiencies resulting from the lack of structured continuity. Without consistent patient-provider assignments or reliable documentation, they often had to begin consultations without context or baseline knowledge. One medical officer from the Surgical Unit explained,

*“Sometimes you come on duty, and a patient says, ‘I’ve already been seen,’ but there’s no documentation. So, you start over. You’re blind. (MO3, healthcare provider)”*

According to the providers, this sense of disconnection was reflected both administratively and clinically, as doctors were unable to develop deeper relationships with their patients or track the outcomes of their interventions.

Several providers further acknowledged that the rotational staffing model contributed significantly to this problem. As one medical officer from the OPD candidly noted,

*“Continuity is hard. I may see a patient on Monday and not again for weeks—if I’m even scheduled to be here. We can’t properly track their progress. (MO2, healthcare provider)”*

Others added that unless they made intentional efforts to follow up on patients, they were unlikely to encounter the same individual twice. Meanwhile, patients sometimes found themselves caught between conflicting opinions or unsure if past decisions were remembered. One recipient mentioned needing to *“remind the doctor what tests I’d done before(SM2, healthcare recipient),”* noting that the burden of maintaining clinical memory had fallen on the patient rather than the system.

The fragmentation of care also manifested in the transition to digital health records. Both groups acknowledged that previous medical histories were not always visible or accessible. A patient participant recalled:

*“They changed the system, and my whole history disappeared. I had to start all over again. (SS4, healthcare recipient)”*

Providers, too, voiced frustration about the electronic system’s limitations. One family medicine doctor explained,

*“If the previous doctor didn’t document well or the notes weren’t entered properly—I was flying blind. (MO2, healthcare provider)”*

### Care without comfort

Participants across the healthcare provider and recipient groups recounted that while medical care was technically delivered in the outpatient department (OPD), it often lacked the emotional depth and safety that are usually associated with genuine healing. The sub-theme “Care without Comfort” captured how the absence of relational continuity, the constraints of time, and the emotional distance imposed by the existing OPD system design made it difficult to establish meaningful connections between patients and their healthcare providers. This aligns with the CFIR Inner Setting domain, particularly organisational culture, implementation climate, and structural conditions that influence the relational quality of care delivery. It also reflects Outer Setting influences in the form of patient expectations for respectful, empathetic, and person-centred care, which contrast with their lived experiences within the system.

Recipients of care, particularly those managing chronic or stress-sensitive conditions, frequently described their clinical encounters as emotionally unsettling. One senior member from the College of Humanities, who lived with hypertension, recounted that she often felt physically worse during hospital visits due to emotional discomfort. She explained that she usually arrived early in the morning, hoping to receive prompt attention, but the unfamiliarity of the doctors she met often heightened her anxiety rather than relieved it. She recalled:

*“I was hypertensive. Anytime I went to the hospital, I got palpitations. If the doctor didn’t calm me, my pressure got worse, (SM6, healthcare recipient)”*

For her, emotional comfort was not a luxury but a clinical necessity. Yet, the OPD’s rotational model meant she frequently met new doctors, none of whom had the time or familiarity to offer the kind of calming presence she needed.

This lack of comfort also affected communication and trust between healthcare providers and recipients. A senior member from the College of Engineering described how difficult it was to open up during appointments. She often felt that her concerns were too rushed or misunderstood. She recounted:

*“Sometimes, if I didn’t know the doctor, I just took the medicine and left. I didn’t even understand what they said (SM2, healthcare recipient)*”

Her statement reflected a broader pattern, where patients avoided asking questions or withheld deeper concerns because the environment felt too impersonal or pressured. The lack of familiarity with providers created not just emotional discomfort, but a barrier to informed decision-making.

From the perspective of healthcare providers, the same emotional gaps were acknowledged, though often from a place of frustration and professional limitation. A medical officer from the Family Medicine unit shared that she understood the emotional needs of her patients but found it nearly impossible to meet them within the constraints of the system. She narrated:

*“People shared more when they trusted the person treating them. That only came with time and continuity, (MO2, healthcare provider)”*

She further went on to acknowledge that, “*with rotational duties, full clinic schedules, and unpredictable patient flow, I rarely saw the same patient more than once*”. Another provider from the OPD expressed similar concerns. He noted that emotional trust and disclosure often took time to develop, but he was usually expected to diagnose, prescribe, and move on within minutes. He further described how emotional depth was routinely sacrificed for speed in the current OPD system.

“*In two minutes, you can’t expect someone to pour out their fears or concerns. You treat the symptoms, not the person (MO6, healthcare provider)”*

According to the participants, the consequences of this disconnection were not merely emotional but also had clinical repercussions. Providers recalled cases where patients withheld important details during consultations, only to disclose them later when rapport had been built by chance. A medical officer who often covered weekend OPD shifts had this to say:

*“When patients didn’t feel safe, they held back. And that change everything—from the diagnosis to the treatment plan (MO1, healthcare provider)”*

This sentiment echoed what recipients themselves had shared that the lack of emotional connection sometimes made them disengage from the care process entirely. For some recipients, this disconnection became a reason to delay or avoid follow-up visits altogether. A senior administrator admitted that she often waited until symptoms worsened before returning to the hospital, simply because she found the experience emotionally draining.

*“It wasn’t just the queue,” she explained. “It was the feeling of going through everything again—with someone new, someone who didn’t know me (SS4, healthcare recipient)”*

A deep emotional gap between patients and their providers was noted across participants’ narratives which was attributed to a lack of compassion resulting from systemic shortfalls that prioritized efficiency over empathy. While providers were aware of the emotional needs of their patients, they were often unable to address them within the constraints of time, staffing, and structure. At the same time, patients found themselves emotionally isolated, unable to connect with those entrusted with their care, and left longing for relational presence rather than procedural treatment.

#### Theme 2: A doctor to call my own.

This theme reflected participants’ hopes, aspirations, and critical evaluations regarding the proposed implementation of a Family Physician Model (FPM) within the university hospital system. For both healthcare providers and recipients, the model represented a departure from fragmented, impersonal OPD care and an opportunity to build continuous, trust-based clinical relationships. These findings are directly linked to the CFIR domains of Intervention Characteristics, Inner Setting, and Process. Specifically, the Intervention Characteristics domain is reflected in participants’ views on the relative advantage, adaptability, and perceived complexity of the FPM compared with the existing OPD system. The Inner Setting domain is evident in discussions around workflow integration, staffing arrangements, communication structures, and readiness for change within the hospital. The Process domain is reflected in participants’ emphasis on planning, stakeholder engagement, phased implementation, and continuous evaluation required for successful adoption of the model. Three sub-themes were generated from this theme namely: “Healing through Continuity”, “Beneath the Optimism”, and “Operationalizing the Vision”.

### Healing through continuity

For many participants, the proposed Family Practice Model symbolized more than an administrative change as it represented a vision of care grounded in memory, relationship, and accountability. In contrast to the episodic, task-driven structure of the current OPD, participants imagined a system where healing could unfold progressively over time, with each encounter deepening the provider’s understanding of the patient, and the patient’s trust in their caregiver. These reflections are linked to the CFIR Intervention Characteristics domain, particularly relative advantage and perceived benefits of the proposed model compared with the existing OPD system.

Healthcare providers, particularly those with experience in long-term care settings, emphasized that clinical excellence was often tied to continuity. One medical officer from the Family Medicine unit noted that repeated contact with a patient enabled her to observe subtle patterns in symptom progression and response to treatment that would likely go unnoticed in isolated consultations. She explained:

*“When I manage someone over time, I start to see the real picture … not just snapshots. I can adjust treatment more precisely, and I know when something’s off even before they say it. (PA1, healthcare provider)”*

This ability to detect early changes, she suggested, could lead to more timely interventions and fewer hospital re-admissions.

From the recipient’s point of view, continuity was described as something that allowed them to become active participants in their own care, rather than passive recipients of hurried decisions. A senior member from the College of Social Sciences reflected on the confidence she felt when her care was overseen by a doctor who already understood her lifestyle, stressors, and medical preferences.

*“I didn’t have to start with ‘what do you do?’ or ‘what’s your history?’ That part was already known, so we could talk about how I was actually feeling. (JS4, healthcare recipient)”*

Several patients also expressed how continuity could support behavioural and lifestyle changes, especially in cases involving chronic disease management. One recipient managing early-stage diabetes stated that regular follow-up with the same doctor helped her stay consistent with diet and exercise recommendations.

*“He didn’t have to remind me every time—he just asked how it was going. I felt like someone was walking the journey with me. (SM3, healthcare recipient)”*

This goes to support that healing was perceived as both physiological and psychological, and required the presence of a consistent guide who would not only prescribe treatments but accompany the patient along the illness journey.

For providers, the continuity promised by the FPM also implied a shift in responsibility from shared institutional care to personal accountability. A medical officer from the Staff Clinic described this as a motivator rather than a burden.

*“If I know these 80 patients are mine, I take responsibility differently. I want to follow through, and I feel bad when they relapse. MO2, healthcare provider)”* She argued that this model would foster a culture of clinical ownership, where providers would become more invested in long-term outcomes rather than short-term fixes.

According to the healthcare providers interviewed, continuity also allowed for nuanced, patient-specific decision-making, especially in borderline cases where protocol alone did not provide clarity. One doctor recounted a case where, based on previous behavioural patterns and lab trends, he delayed starting a patient on antihypertensives and focused instead on lifestyle counselling successfully.

*“If I didn’t know her history, I would’ve started medication. But I had seen her make changes before, and I trusted she’d do it again. And she did. (MO4, healthcare provider)”*

This account highlighted how relational familiarity could lead to more personalized, less invasive care, often aligning more closely with the patient’s own goals.

Participants also believed that continuity through the FPM would help minimize unnecessary duplication of both tests and instructions while creating a feedback loop for improvement. As one recipient put it,

*“When the same person sees you, they also learn from their own decisions. They remember what worked last time, and they don’t keep repeating things. SM6, healthcare recipient)”*

Overall, “Healing through Continuity” captured a collective longing for care that evolves, remembers, and responds. Participants did not imagine a perfect system, but they envisioned one where relationships replace rotation, and where physicians and patients grow together through cycles of interaction, adaptation, and trust. This sub-theme thus illuminated continuity as a clinical asset, a psychological comfort, and a moral anchor, which are regarded as essential ingredients for restoring the integrity of everyday care in the hospital setting.

### Beneath the optimism

While participants spoke positively about the potential of the Family Practice Model to transform care, their enthusiasm was often accompanied by practical concerns, hidden uncertainties, and thoughtful caution. This sub-theme, “Beneath the Optimism”, captured the layer of hesitation that underpinned the supportive narratives. These reflections are linked to the CFIR Intervention Characteristics domain (feasibility, complexity, and adaptability) as well as the Inner Setting domain (readiness for implementation, staffing capacity, and infrastructure constraints). While participants strongly supported the FPM in theory, their experiences with the current healthcare system led to pragmatic concerns about its implementation. Patients and providers alike questioned whether the model could succeed without addressing deeper structural issues in care delivery.

From the perspective of healthcare providers, concerns centered around workload, feasibility, and system preparedness. Several medical officers reflected on how the model might sound ideal in theory but would face serious challenges in implementation. A medical officer from the Emergency Unit shared:

*“You assign me 100 patients, and 20 show up on one day … what do I do? I’m already stretched. (MO1, healthcare provider)”*

Her statement underscored the fear of overburdening frontline staff, especially in a hospital already grappling with high patient volume and limited personnel.

Another provider from the General OPD echoed this anxiety, stating that:

*“… continuity sounds good, but if the doctors don’t have protected time for follow-up, then it’s just another rotation with a new label. (MO3, healthcare provider)”*

The above quote highlighted the risk of the model being superficially adopted, without the underlying policy and staffing support needed to sustain it. The narratives shared by the participants point to the fact that for the model to work, the institution would need to redesign schedules, define manageable patient loads, and support documentation continuity.

On the recipients’ side, optimism was counterbalanced by concerns of flexibility, autonomy, and access. While many welcomed the idea of a consistent physician, several emphasized the importance of having choice and recourse within the model. A senior member from the Engineering Faculty put it plainly:

*“If I already know a doctor, I’m comfortable with, let me choose. If it doesn’t work, let me change. Don’t lock me in. (SM2, healthcare recipient)”*

For her, the model’s success would depend not just on assigning patients to physicians, but on creating space for relationships to evolve and, when necessary, to be adjusted.

Others feared that their assigned physician might be unavailable during emergencies, or that continuity could result in delayed access to urgent care. One patient shared,

*“What if my family physician is off duty and I have a crisis? Will I be forced to wait or go through someone else who doesn’t know me? (JS1, healthcare recipient)”*

This concern reflected a deeper tension: while continuity brought comfort, it also risked limiting patient mobility and flexibility if not properly designed.

Additionally, both groups of participants stressed that infrastructure and institutional systems must evolve alongside the model. Providers called for robust documentation systems, clear triage protocols, and consistent follow-up mechanisms. As one doctor noted:

*“Assigning doctors is not enough. You need reminders, records, ways to track people and follow them up properly. (MO6, healthcare provider)”*

This emphasis on operational integration revealed how optimism about the FPM was grounded in realism. Without changes to information systems, staffing models, and training, participants feared the model might fail under the weight of good intentions.

Simultaneously, participants articulated a collective vision for the FPM as a catalyst for transformative change in care delivery. However, they emphasized that realizing this potential would require redesigned systems that maintain continuous access and adaptable frameworks for local customization. A provider in Family Medicine expressed this sentiment with cautious hope:

*“It can work. But don’t rush it. Start small, learn, and expand gradually. If you dump the whole thing at once, it won’t survive. (MO2, healthcare provider)”*

The idea of phased implementation resonated strongly with both groups. Participants recommended beginning with staff clinics, student health services, or chronic care patients, where patient loads were more defined and outcomes easier to measure. One recipient suggested,

*“Start with the people who already come often — diabetics, hypertensives, antenatal cases. That way you can test it with the people who actually need follow-up. (SM3, healthcare recipient)”*

### Operationalizing the vision

While earlier reflections captured participants’ emotional investments and cautionary concerns surrounding the Family Practice Model (FPM), this sub-theme, “Operationalizing the Vision”, shifted decisively toward constructive engagement. Participants spoke as co-designers, offering specific, context-driven proposals on how the model might be successfully embedded into the hospital’s healthcare infrastructure. These findings are closely linked to the CFIR Process domain, particularly planning, engaging, executing, and reflecting and evaluating, as well as elements of the Inner Setting domain relating to workflow integration and communication structures. Thus, providers and recipients alike approached the FPM not only as beneficiaries but as active architects of its future.

Among healthcare providers, conversations centered on the structural, procedural, and clinical adaptations needed to make the model viable. A medical officer in Family Medicine suggested that success depended on integrating the FPM into existing clinical structures and not attempting to overhaul the system all at once. She narrated:

*“Don’t reinvent the wheel. Start where continuity already happens … chronic care, antenatal. These patients are already used to follow-up,” she said (MO2, healthcare provider).*

Others emphasized the importance of protecting time and streamlining documentation. A provider in the OPD stressed the need for workflow reconfiguration:

*“If we’re meant to follow up with specific patients, then carve out a window for that. Otherwise, it becomes another good idea that dies in traffic. (MO3, healthcare provider)”*

This drew attention to the need for institutionally sanctioned scheduling, where dedicated family physician time slots would allow for in-depth engagement. One striking idea emerged from a senior clinician in the Emergency Unit, who advocated for a micro-team model, where a small group of doctors shared responsibility for a defined patient pool.

*“We don’t have enough staff for one-on-one pairings. But what if you assigned a group of patients to a trio of doctors? That way, familiarity builds but the load is shared. MO1, healthcare provider)”*

Providers also raised the need for technical systems that supported relational continuity. As one Family Medicine physician noted:

*“We need a tracking system … alerts, digital folders, continuity indicators. If I’m someone’s family doctor, the system should remind me when they’re due. (PA2, healthcare provider)”*

These proposals framed the model as one that would require technical integration and design coherence, not just role assignment.

Healthcare recipients provided crucial frontline insights into the practical implementation of the new model. While they agreed with prioritizing high-need populations, their emphasis was on ensuring clarity, accessibility, and flexibility for patients within the new structure. A recipient from the College of Science, managing diabetes, proposed a clear onboarding process:

*“If you’re assigning me to someone, tell me. Explain who they are, what they’ll do, and when I can reach them. (SM3, healthcare recipient)”*

Another recipient, a senior staff member in Administration, stressed the need for visible infrastructure that supports the idea of a personal physician. She stressed:

*“Give us a card or ID with our doctor’s name. Let it show in the system when we book. Make it feel official. (SS2, healthcare recipient)”*

For her, the FPM would not be fully trusted unless it was institutionalized in both language and logistics.

With regards to rollout strategy, patients advocated for pilots and feedback loops. One patient participant suggested launching the model within the staff clinic or student health services, where patient populations were easier to monitor, and lessons more straightforward to learn.

*“Start small, test, adjust, and expand. Otherwise, it becomes too much too fast, (JS1, healthcare recipient)”*

Recipients also emphasized the need for reassignment flexibility beyond the initial provider choice. While previous discussions had centered on patient preferences at the outset, participants’ narratives in this study focused on resolving provider-patient mismatches that emerged over time. Their accounts highlighted how therapeutic relationships could deteriorate despite careful initial matching, whether due to changing circumstances, incompatible communication styles, or treatment approaches that no longer aligned with evolving patient needs. A participant recounted:

*“If the relationship isn’t working, I should be able to request a change … without fear or long processes. (SS1, healthcare recipient)”*

These narratives reflected participants’ desire for a healthcare system that valued not only thoughtful initial pairing but also mechanisms to readjust relationships when necessary, acknowledging the dynamic nature of effective patient-provider connections.

## Discussion

This study examined the perspectives of healthcare providers and recipients on the existing OPD system at the University Hospital, KNUST and the prospective shift to FPM. Findings revealed significant discontinuity and emotional disconnect in the current model, marked by rushed, fragmented, and impersonal care, against which participants expressed strong aspirations for sustained, relationship-centered care. Using the Consolidated Framework for Implementation Research (CFIR) as an interpretive lens, these findings highlight how contextual factors across the Inner Setting, Outer Setting, Process and Intervention Characteristics domains collectively shape the feasibility and acceptability of the proposed model.

The study’s findings align with global evidence that fragmented care systems contribute to patient dissatisfaction, ineffective chronic disease management, and provider burnout [8,31]. Within the inner setting, participants described weak organizational processes, including poor documentation practices, inadequate communication channels, and frequent provider rotations that disrupted continuity. These characteristics collectively defined what this study captured under the theme “Therapeutic Disconnection. Similar to investigations in South Africa [32] and Nigeria [33] participants highlighted the limitations of rotational physician models, especially for individuals with long-term conditions. In contrast to high-income country studies that primarily emphasize the clinical benefits of continuity (such as decreased hospital admissions and enhanced treatment adherence), our work reveals several context-specific challenges encountered within both the outer setting (limited resources, patient needs for flexible care arrangements) and the inner setting (workload pressures, inadequate staffing, and space constraints). Concerns about potential implementation challenges, including provider-patient mismatches, resource constraints, and workforce shortages necessitate an adaptation of the FPM that is attuned to local realities rather than a “hook, line, and sinker” adoption of frameworks developed in high-income settings. This further echoes the arguments of Phillips and others [34] and reinforces the need for a comprehensive restructuring of clinical workflows to ensure proactive and sustained care.

The implications of these findings are significant. A transition to an FPM would require a comprehensive restructuring of clinical workflows at the University Hospital of KNUST. This includes redesigning appointment systems to ensure provider consistency, enhancing documentation protocols, and developing effective communication mechanisms for when provider transitions occur. These represent critical components of the process domain of CFIR, which reflects the need for planned, systematic, and well-monitored implementation processes. The findings thus call for strong institutional commitment. In view of this, hospital administrators and policy makers must establish formal guidelines for FPM implementation that include standards for provider-patient assignment, continuity protocols, evaluation mechanisms and reallocation of resources (staffing adjustments, space reorganization, and investments in information technology) to facilitate seamless care coordination. Nationally, these insights are expected to bolster ongoing efforts to strengthen primary healthcare, serving as a cornerstone for Ghana’s push towards universal health coverage [35,36]. Within the intervention characteristics domain, participants viewed the FPM as advantageous because of its relative benefit, improved continuity, trust, and satisfaction, though they acknowledged potential challenges relating to adaptability and cost.

The integration of nurse practitioners and community health workers into FPM teams could also help alleviate physician shortages and improve service accessibility, particularly in resource-constrained settings. Drawing on lessons from Ghana’s Community-Based Health Planning and Services initiative [37], task shifting and the integration of team-based care models appear promising. Thus, incorporating nurse practitioners and community health workers into FPM teams, especially in resource-constrained settings may mitigate physician shortages and enhance service accessibility, ultimately improving the continuity and quality of care [32,33]. Furthermore, the transition to an FPM would require a comprehensive restructuring of clinical workflows to include redesigning appointment systems to ensure provider consistency, enhancing documentation protocols and developing effective communication mechanisms when provider transitions occur.

Participants believed that assigning a dedicated provider would streamline care by eliminating repeated history-taking while fostering trust and emotional security. This aligns with the CFIR’s characteristics of individuals domain, emphasizing provider commitment, motivation, and self-efficacy as enablers of effective implementation. This perspective is supported by Dossa et al. [38] study, which found that continuous provider relationships yielded improved treatment adherence and patient satisfaction. Importantly, participants emphasized their desire for enduring, trust-based relationships, which are core expectations of effective healthcare delivery in the Ghanaian cultural context [39]. Sentiments from participants underscored the importance of strategic planning, stakeholder engagement, and the formulation of clear protocols that balance continuity with flexibility, key elements within the process domain of CFIR. Given this, unique challenges such as accommodating academic schedules and balancing teaching with clinical care must be addressed in university hospital settings. These considerations echo the findings of Amankwah et al. [40] and underscore the point that effective FPM implementation must be specifically adapted to the institution’s diverse and complex context.

In the area of education, there is a clear need for curriculum revisions in medical and nursing programs to incorporate family medicine principles, with an emphasis on long-term relationship management, comprehensive care coordination, and effective communication. Regular faculty development and interprofessional education initiatives will further support the transition toward a collaborative, continuity-focused model. This aligns with the outer setting domain, as broader educational and policy environments influence readiness for change. Such an approach is consistent with the World Health Organization’s call for “transformative education” aimed at developing primary healthcare-oriented workforce [41] ensuring that healthcare providers are clinically proficient to provide patient-centered care.

At the policy and leadership level, our findings underscore the critical need for national strategies that actively incentivize the retention of primary healthcare professionals. These can include financial bonuses, opportunities for career development, professional recognition, early promotion, provision of staff accommodation, and supportive working conditions [35,42,43]. Additionally, reforming reimbursement structures under Ghana’s National Health Insurance Scheme to reward continuity of care and clinical outcomes, rather than merely episodic service delivery could further support the successful integration of the FPM. Such policy adjustments align with the outer setting domain, that can address and drive system-level changes and external incentives with the principles of equity and efficiency for implementation success.

Future research should explore pilot studies that examine adequate operational capacity, assess the program’s cost-effectiveness, and evaluate the role of digitization in care coordination. Moreover, research should investigate how the FPM can be effectively linked to Ghana’s broader Universal Health Coverage goals, as outlined in the 2020–2030 Health Sector Strategy. A robust FPM implementation has the potential to reduce redundant testing, improve medication adherence, and ease referral pressures on tertiary care facilities as these objectives are central to sustainable healthcare financing. Additionally, by centering care on established provider-patient relationships, the FPM could significantly enhance equity, particularly among vulnerable populations such as elderly staff and low-income dependents.

### Limitations

This study is not without limitations. First, the participant pool was limited to healthcare providers and university staff who accessed OPD services, which may not fully capture the experiences of community members who also utilize the facility. Next, findings may reflect institutional perspectives more strongly than those of the general public. Second, as with most qualitative designs, the findings are context-specific and not intended for statistical generalization. Third, despite efforts to encourage openness, participants may have provided socially desirable responses given the institutional setting and the topic’s sensitivity. Finally, while data saturation was achieved, expanding future research to include focus groups or mixed-method approaches could provide additional perspectives and enhance external validity. These limitations notwithstanding, the study offers valuable, contextually grounded insights that can inform the early design and piloting of a Family Practice Model within similar institutional healthcare settings.

## Conclusion

Stakeholder perspectives indicate a distinctly favourable view of the FPM in contrast to the existing, fragmented outpatient model. While continuing care clearly holds promise for improving clinical outcomes and patient satisfaction, successful implementation will require significant adaptations to fit the local context. Recommendations include piloting the FPM at the University Hospital, KNUST with iterative evaluation, revising educational curricula to address gaps in primary care training, and adopting policies that financially and structurally incentivize continuity. Future pilot studies could assess specific measurable outcomes such as reduced hospitalization and readmission rates, improved chronic disease control indicators (e.g., blood pressure, glucose levels), increased patient trust and satisfaction scores, and provider workload balance to determine the model’s feasibility and impact. As Ghana strives toward universal health coverage, integrating relationship-centered models like the FPM may offer a sustainable pathway to enhancing primary healthcare quality, reducing inefficiencies, and promoting health equity across diverse populations. A structured booking system should be introduced to manage patient flow effectively and minimize long waiting times.

In summary, this study reveals that a well-adapted FPM holds promise as a means to address the current system’s deficiencies by enhancing continuity and fostering trust-based relationships. Integrated, context-sensitive reform in clinical practice, education, leadership, and policy is essential to realize the full potential of the FPM, thereby contributing to more equitable and efficient healthcare delivery and advancing Ghana’s universal health coverage goals.

## Supporting information

S1 Appendix Interview guide for participants. Semi-structured interview guide for both healthcare providers and recipients used for data collection.
(DOCX)

S2 Appendix SRQR_checklist. Standards for reporting qualitative research checklist.
(DOCX)
