# Supplementary material for: Exploring patient and provider perspectives on outpatient care and the family practice model at a university hospital in Ghana
Source: PLoS One. 2026 Sep 9;21(9):e0327277. doi: 10.1371/journal.pone.0327277 (PMC13557406; doi:10.1371/journal.pone.0327277)
Supplement: Supplementary file 1 [file pone.0327277.s005.pdf]

RESPONSE TO REVIEWERS

First reviewer

Comment	Response
1.Please ensure that your manuscript meets PLOS ONE's style requirements, including those for file naming.	We appreciate the reminder regarding PLOS ONE's formatting and style requirements. The entire manuscript has been reviewed and reformatted to comply with the PLOS ONE style guidelines. Specifically:<br>The main text, tables, and references have been formatted according to the PLOS ONE sample templates provided.<br>The title page, including author names, affiliations, and corresponding author details, now conforms to the required PLOS ONE format.<br>File names have been revised to meet PLOS ONE naming conventions.
2. We note that your Data Availability Statement is currently as follows: All relevant data are within the manuscript and in Supporting Information files.<br><br>Please confirm at this time whether or not your submission contains all raw data required to replicate the results of your study. Authors must share the "minimal data set" for their submission. PLOS defines the minimal data set to consist of the data required to replicate all study findings reported in the article, as well as related metadata and methods (https://journals.plos.org/plosone/s/data-availability#loc-minimal-data-set-definition).<br><br>For example, authors should submit the following data:<br><br>- The values behind the means, standard deviations and other measures reported;<br><br>- The values used to build graphs;<br><br>- The points extracted from images for analysis.	Thank you very much. We confirm that all relevant data required to replicate the findings of this qualitative study are contained within the manuscript and the accompanying Supporting Information files (S1–S3 Appendices).

Authors do not need to submit their entire data set if only a portion of the data was used in the reported study.<br><br>If your submission does not contain these data, please either upload them as Supporting Information files or deposit them to a stable, public repository and provide us with the relevant URLs, DOIs, or accession numbers. For a list of recommended repositories, please see https://journals.plos.org/plosone/s/recommended-repositories.<br><br>If there are ethical or legal restrictions on sharing a de-identified data set, please explain them in detail (e.g., data contain potentially sensitive information, data are owned by a third-party organization, etc.) and who has imposed them (e.g., an ethics committee). Please also provide contact information for a data access committee, ethics committee, or other institutional body to which data requests may be sent. If data are owned by a third party, please indicate how others may request data access.	
3. We note that this data set consists of interview transcripts. Can you please confirm that all participants gave consent for interview transcript to be published?<br><br>If they DID provide consent for these transcripts to be published, please also confirm that the transcripts do not contain any potentially identifying information (or let us know if the participants consented to having their personal details published and made publicly available). We consider the following details to be identifying information:<br><br>- Names, nicknames, and initials<br><br>- Age more specific than round numbers<br><br>- GPS coordinates, physical addresses, IP addresses, email addresses<br><br>- Information in small sample sizes (e.g. 40 students from X class in X year at X university)	Thank you. Please we confirm that all participants provided written informed consent prior to their participation, including consent for their anonymized interview transcripts to be used and published for research purposes.<br><br>The transcripts have been carefully de-identified to maintain confidentiality and privacy. They do not contain any potentially identifying information such as names, nicknames, initials, specific ages, physical or email addresses, GPS coordinates, visit or interview dates, or ID numbers.

- Specific dates (e.g. visit dates, interview dates)<br><br>- ID numbers	
**Additional comments:**	
The topic is interesting and important to the health sector at large to learn from existing practice and experience. The study could be very useful to inform policy.<br>However, there are fundamental issues with the methodology and authors will have to address them.<br>Some of the issues are as follows:	Please we appreciate your observations on the methodological aspects of our study. We have addressed these concerns comprehensively in the revised manuscript.
1. Study adopted a limited number of study participants for a purposive sampling without any justification nor adequate criteria. It is not convincing that the findings from such a limited study can support the conclusion and recommendations made for policy and practice.	Thank you for this comment. We wish to clarify that the participants selected were people who constituted the hospital's core and had direct experience with the OPD system whose opinions would be useful in piloting the FPM before extending it to surrounding communities.<br>(see: participant recruitment, page 8-10)
2. Inadequate justification for choosing such a study design to assess a whole OPD system of a unique hospital facility serving a university community and other 4-5 general communities. Instead of choosing a more representative study design (including participants) to support a generalization.	Thank you. We wish to clarify that our main aim for choosing this study design was to capture the in-depth views and insights of key stakeholders for which the introduction of FPM would be piloted. (see study design section, page 7).
3.Unfortunately, the criteria for selection of participants is unclear - especially patients, it's not clear why only staff of the university were chosen. What about the larger general communities that authors claim are served by the hospital?	Thank you. Please our study focused on university staff (senior members, senior staffs and junior members) because they represent the hospital's core and most consistent outpatient population, whose experiences typify the dominant service patterns at the University Hospital, KNUST. Given that the Family Practice Model (FPM) is being considered for initial implementation within the university hospital system, targeting this group allowed for a controlled exploration of feasibility and acceptability before potential extension to the wider surrounding communities.

	(see Participant recruitment section, page 8-10).
it's emphasising the limitation that the university staff may be receiving different level of service, may be preferentially different from the general community members. What was the interest or the purpose of the study, is it to satisfy only staff patients? meanwhile we know that staff OPD is clearly different from the general public. Personally, I have visited the facility several times and I have a course to believe that I am treated differently at the OPD compared to others who are staff of the university.	The purpose of this study was not to assess satisfaction among staff patients alone, but rather to explore the feasibility, acceptability, and contextual fit of the proposed Family Practice Model (FPM) within the University Hospital, KNUST. The decision to focus on university staff was intentional and based on two reasons. First, the FPM is intended for pilot implementation within the hospital's core outpatient population primarily university staffs, before potential scale-up to the general public. Second, university staff represent a stable and accessible population with consistent service utilization patterns, making them appropriate for initial qualitative exploration of the model.<br>We agree that staff may experience care dynamics that differ from those of the general public, and this has been acknowledged as a limitation in the revised. Refer to Page 38, lines 779-791.
4.A cross-sectional study involving questionnaire administration would have helped improve data collection? Better still, with the same qualitative approach, couldn't the use of focus groups with some community patients/members have helped to bring their perspective to bear?<br>Meanwhile, no evidence is presented that participant selection achieved any theoretical saturation with respect to the emerging themes or findings expected from a qualitative study.	Thank you for this suggestion. However, the study's qualitative design was deliberately chosen to explore, in depth, the nuanced experiences and expectations of key stakeholders for which the FPM introduction will be piloted on.<br>We acknowledge that focus group discussions could have enriched the dataset, individual in-depth interviews were prioritized to ensure participants could freely express potentially sensitive or critical views about existing care systems without peer influence or hierarchical pressure, particularly relevant in a university hospital setting where providers and recipients often share institutional affiliations.<br>Regarding theoretical saturation, data collection and analysis were conducted concurrently, and recruitment continued until no new themes or insights emerged. This point of thematic saturation was reached after the 25th interview, consistent with guidance for qualitative inquiry of this nature (Wultich et al., 2024). Refer to the methods section on page 11, lines 231-233).

5.Again, no limitations enumerated openly for readers to appreciate that authors have acknowledged those that were beyond their control - obviously there are several of such with this kind of study design that the authors failed to indicate them.	We appreciate this observation and agree that stating the study's limitations strengthens the transparency and interpretability of our findings. A dedicated paragraph has been added to the Discussion section to acknowledge methodological and contextual limitations, such as restricted participant scope, potential social desirability bias, and limited generalizability inherent to qualitative inquiry. Refer to the Limitations section on page 38, line 779-791.
6. Also, these comments should be considered for revision:<br>Abstract:<br>o Keywords too loaded, should consider standard keywords in the discipline using less of 3 words phrases.<br>o background of abstract too lengthy, it should be more focused and concise.<br>o avoid use of acronyms in abstract FPM for first time use	Thank you for your feedback. Please we have revised the abstract to make the background more concise and focused on the study purpose, avoided the use of acronyms on first mention, and replaced overly specific multi-word phrases in the keywords with standardized, discipline-appropriate terms to enhance discoverability. See Abstract section on page 2).
4. The manuscript has been presented in an intelligible fashion and written in standard English. However, the authors should consider reviewing some grammatical errors. For example, the first paragraph in the introduction section with reads "Primary healthcare is widely recognized as the cornerstone of effective health systems, as it substantially improves access to essential services, strengthens disease prevention, and supports coordinated care over" seems to be incomplete and might confuse the readers.	Thank you. The incomplete sentence in the Introduction has been corrected to improve clarity and flow. The revised sentence now reads:<br>"Primary healthcare is widely recognized as the cornerstone of effective health systems, as it enhances access to essential services, strengthens disease prevention, and supports coordinated care throughout a person's life course"<br>Additionally, the entire manuscript has been carefully proofread to address minor grammatical and stylistic issues for enhanced readability (see introduction, page3).

Second Reviewer

**Comment**	**Response**
1. Clarify Study Duration (Critical): Please confirm and correct the discrepancy between the study duration stated in the Abstract (April to May 2024) and the	Please, we have reviewed the manuscript and confirm that the correct study duration is

Methods section (September 2023 to May 2024). This is a technical error that must be resolved.	September 2023 to May 2024. This period reflects the entire timeline of data collection and analysis. (see: data collection and analysis; refer to page 10, lines 203-205).
2. Integration of CFIR in Discussion: While the CFIR is effectively introduced in the Methods, the Discussion section should be structured to explicitly link the identified themes and sub-themes back to the relevant CFIR domains (e.g., Inner Setting, Characteristics of Individuals, Intervention Characteristics). For instance:<br>-The "Therapeutic Disconnection" theme relates directly to a weak Inner Setting (poor documentation, rushed consultations).<br>- The "Beneath the Optimism" sub-theme clearly relates to Outer Setting (patient needs for flexibility) and Inner Setting (workload, resource availability).<br>- Strengthening this discussion will maximize the manuscript's contribution to implementation science.	Please we have revised the Discussion section to clearly map each major theme and sub-theme to the relevant CFIR domains to enhance conceptual clarity and strengthen the study's contribution to implementation science. Refer to the discussion section on page 34-38.
1. Sampling Justification: The authors note that care recipients were university staff. While purposive sampling is justified, briefly explain why this specific group of care recipients (university staff) was chosen over a broader sample from the community or general hospital users. Does this reflect a targeted initial implementation phase or a specific population served by the hospital?	Please the selection of university staff as care recipients was intentional and based on the hospital's primary service population. The proposed FPM is expected to be piloted internally for university staffs before broader community implementation. This purposive sampling thus ensured relevance to the hospital's operational context and the study's exploratory objectives.
2. Illustrative Quotes: The manuscript includes excellent quotes (e.g., "You don't have time to breathe. You just keep going..."). Please ensure all main sub-themes (e.g., "Care on the clock," "Fragmented care," "Care without comfort," "Healing through continuity") are supported by direct, clearly cited quotes within the results section for maximum impact and transparency.<br><br>3. Future Research/Pilot Testing: The conclusion mentions the need for pilot testing. It would be beneficial to briefly suggest specific, measurable outcomes that future pilot studies could use to assess	Thank you for this kind suggestion. However, we have ensured that each main sub-theme, including "Care on the clock," "Fragmented care," "Care without comfort," and "Healing through continuity" is supported by direct, clearly cited illustrative quotes from participants. Refer to Pages 19-33.<br><br>Thank you. We have revised the conclusion to specify measurable outcomes that future pilot studies could employ to assess the feasibility and impact of the Family Physician Model (FPM).

FPM feasibility and impact (e.g., reduced hospitalization rates, improved chronic disease control, change in trust scores).	These include indicators such as reduced hospitalization and readmission rates, improved chronic disease control (e.g., blood pressure and glucose monitoring outcomes), enhanced patient satisfaction and trust scores, and provider workload balance. Refer to the conclusion section on page 39, lines 801 to 804.
