# Supplementary material for: Exploring patient and provider perspectives on outpatient care and the family practice model at a university hospital in Ghana
Source: PLoS One. 2026 Sep 9;21(9):e0327277. doi: 10.1371/journal.pone.0327277 (PMC13557406; doi:10.1371/journal.pone.0327277)
Supplement: Supplementary file 2 [file pone.0327277.s006.pdf]

**Exploring patient and provider perspectives on outpatient care and the family practice model at a university hospital in Ghana**

**Response to Review Comments**

| Number | Comment/suggestions | Response to comments | Page |
|---|---|---|---|
| 1. | Expert purposive sampling could have been used for healthcare providers based on their work or where they work. | Thank you for the comment. However, we wish to clarify that we used purposive sampling with a maximum variation strategy to recruit healthcare providers, specifically targeting medical officers and physician assistants with at least one year of experience in the hospital OPD. This ensured inclusion of information-rich participants with direct experience of the system under study. While expert purposive sampling could also have been appropriate, our approach was chosen to capture a broader range of provider perspectives across roles and units rather than limiting selection to a narrower expert subgroup. | 171 – 176 |
| 2. | Healthcare recipients could have been recruited through convenience sampling to reach several university staff who were accessing healthcare at that time. Non-university staff could be excluded. | That is very thoughtful. However, healthcare recipients were purposively recruited among university staff who had accessed OPD services within the past year. This was intentional to ensure participants had relevant, recent experience with the system under study. We opted against convenience sampling to avoid capturing only those present at the health facility at the time of data collection, which could have introduced selection bias and limited variation in experiences. Restricting inclusion to university staff was | |

| | | | |
|---|---|---|---|
| | | also deliberate, as they represent a stable and consistent user group of the hospital's OPD services, which was important for the objectives of this study. | |
| 2 | Did you get a translator for the Twi transcription? | Thanks for the comment. Clarification on the process has been provided in the revised manuscript as follows:<br>Interviews conducted in Twi were transcribed and translated into English by bilingual members of the research team who were fluent in both languages. The accuracy of the translations was validated by a Twi language expert, and all transcripts were cross-checked against the original audio recordings to ensure fidelity and accuracy. | 206-209 |
| 3 | Why did the data collection take so long? | Thank you for this observation. We wish to clarify that the data collection period, from September 2023 to May 2024, reflected the practical and methodological demands of the study rather than delays in the research process. The study involved two stakeholder groups: healthcare providers and university staff who had accessed OPD services. Many participants had demanding and variable clinical, administrative, and academic schedules; therefore, interviews had to be arranged at times and locations convenient to them to minimise disruption to their duties.<br><br>In addition, data collection was conducted concurrently with transcription, translation where necessary, and preliminary analysis. This iterative process | 203, 233-234 |

| | | | |
|---|---|---|---|
| | | allowed the research team to refine interview probes, compare emerging insights across participant groups, and continue recruitment until thematic saturation was achieved. The extended period was therefore necessary to ensure adequate participant diversity, depth of inquiry, reflexivity, and methodological rigour. | |
| 4 | Were the pretests conducted among healthcare providers or recipients? How many of each group of participants were pretested before the study | Thank you for this important observation. The interview guides were pretested among both participant groups prior to the main study. Specifically, the pretest involved seven healthcare providers and seven healthcare recipients from a similar hospital setting.<br>Their feedback was used to improve the clarity, sequencing, relevance and flow of the questions as the overall focus of the guide remained unchanged. | 220 - 223 |
| 5 | Your reason for using a hybrid approach to your thematic analysis while using a model is not clear | Thank you for this comment. We adopted a hybrid thematic analysis approach to ensure that the analysis was guided by the conceptual model without being constrained by it. The model provided a deductive structure for examining issues central to the study, while inductive coding allowed unanticipated and context-specific insights to emerge from participants' narratives. This approach enhanced analytical rigour by ensuring that the findings were both theoretically informed and grounded in the empirical data. | 240-245 |
| 6 | I am not sure of how you are analyzing your work. I | Thank you. However, we wish to clarify that our analysis was | 339, 351-357, 365- |

| | | | |
|---|---|---|---|
| | initially thought you used the main constructs of the Consolidated Framework for Implementation research as your main themes (deductive), and the sub-themes were aligned with the study's constructs (inductive). However, I cannot find any construct of the model you have chosen; all I see are the inductive themes you have generated. The themes' connections to the framework are not clear. | guided by a hybrid approach rather than using CFIR constructs as preset themes. CFIR informed data collection, coding, and interpretation, but themes were derived inductively from participants' accounts to preserve depth and context. To address the concern, we have strengthened the Results section by explicitly linking the themes to CFIR constructs. For example, Theme 1 to the Inner Setting (and relevant Outer Setting influences) and Theme 2 to Intervention Characteristics, Inner Setting, and Process domains, making the alignment with CFIR more transparent. | 367, 397-402, 458-462, 525-532, 542-544, 605-608, 669-672. |
| 7 | None of the participants' quotes was referenced. | Thank you for this observation. We have revised all quotations in the results section to clearly indicate the source of each statement, specifying whether it was provided by a healthcare provider or a healthcare recipient. | 373-726 |
| 8 | Be consistent with the spacing between the numbers and percentages | We have reviewed the manuscript and corrected all instances to ensure consistent formatting of numbers and percentages throughout the text. | Page 1 -47 |
| 9 | The table did not specify the population whose themes and subthemes were generated | Thank you. We have revised Table 3 to clearly indicate that the themes and sub-themes were derived from both healthcare providers and healthcare recipients (university staff) who participated in the study. | 339 |
| 10 | When was data saturation achieved for each of the two groups of participants? | Data saturation was reached at different points for the two participant groups. For healthcare providers, saturation | 194 - 200 |

| | | | |
|---|---|---|---|
| | | was achieved after the 8th interview, while for healthcare recipients, it was reached after the 17th interview. However, interviews continued up to 9 providers and 19 recipients to ensure full coverage and confirm that no new themes or perspectives were emerging across either group. | |
