# Supplementary material for: Exploring patient and provider perspectives on outpatient care and the family practice model at a university hospital in Ghana
Source: PLoS One. 2026 Sep 9;21(9):e0327277. doi: 10.1371/journal.pone.0327277 (PMC13557406; doi:10.1371/journal.pone.0327277)
Supplement: Supplementary file 3 [file pone.0327277.s001.docx]

**FAMILY PRACTICE MODEL OF HEALTHCARE DELIVERY AT A UNIVERSITY HOSPITAL**

**UNIVERSITY STAFF (RECIPIENTS OF CARE)**

**Instructions**

We are currently embarking on a research project which seeks to explore university staff experiences on seeking healthcare at the out-patients’ department of the university hospital, as well as their perceptions and expectations of a family practice model of healthcare. All responses provided will be anonymized and the information provided will be treated confidentially. You are at liberty to withdraw from the study at any point in time and will not be penalized in any form for doing so. Thanks for agreeing to be part of this study.

**Socio-demographics**

1. Age/ Date of birth:
2. Gender (Male/ Female/ Other):
3. Marital Status:
4. Number of children/dependents who seek for healthcare at this facility:
5. College/Faculty/Department/Unit within KNUST:

**Preliminary Questions**

1. How long have you (and your family) been seeking for healthcare in this hospital?
2. What are your experiences on seeking for healthcare at the out-patient department (OPD) of this hospital?

Further probes: Patient-doctor relationship (eg. Communication behaviour, attitudes and behaviours), information flow, waiting time for consultation, responsiveness of healthcare providers, etc

3. What are your views/ opinions about the current OPD system whereby patients and/or their families do not have a specific doctor assigned to them (and are seen by any doctor on duty)?

Prompts: Advantages and disadvantages/challenges of the current system

4. To what extent are you satisfied with the current OPD system whereby patients and/or their families do not have a specific doctor assigned to them (and are seen by any doctor on duty)?
5. In your opinion, what are some of the measures that can be put in place to improve the care of patients at the OPD of this hospital?

**Brief Information about the Family Practice Model of Healthcare**

Family practice model of healthcare is a system whereby a patient and their family members are seen by one doctor. This system has been shown to be cost-effective and efficient in providing quality and continuous healthcare for patients and their families.

As part of efforts to improve the efficiency and effectiveness of OPD services and healthcare in general at this hospital, we want to introduce the family practice model of care and would be grateful to have your input on your perceptions and expectation of this model.

**Main Study Questions**

1. What is your opinion on the introduction of the family practice model (FPM) of care in the OPD system of this hospital?
2. If this system is introduced, would you prefer to choose your doctor or you would want to be allocated one by the hospital? Please provide a reason for your choice.
3. What factors should be considered in the selection of a family practice? / What factors would you consider in your choice of family practice?

Prompts: gender, age, attributes/character, competencies, etc.

4. What advantages do you perceive in having a family practice model of OPD care compared to the current system whereby you and your family do not have a specific doctor assigned to them (and are seen by any doctor on duty)?

Prompts: Advantages of FPM to patients, families and healthcare professionals.

5. What challenges or drawbacks do you anticipate in the proposed family practice model of OPD care?

Prompts: Delays in accessing care, prolonged waiting times, insufficient competencies in caring for all body systems and all categories of family members, etc.

6. In what ways can the above-mentioned challenges be addressed?
7. Please do you have any other comments that were not addressed by the questions?

**Thanks for participating!**

**FAMILY PRACTICE MODEL OF HEALTHCARE DELIVERY AT A UNIVERSITY HOSPITAL**

**HEALTHCARE PROFESSIONALS (PROVIDERS OF CARE)**

**Instructions**

We are currently embarking on a research project which seeks to explore university staff experiences on seeking healthcare at the out-patients’ department of the university hospital, as well as their perceptions and expectations of a family practice model of healthcare. All responses provided will be anonymized and the information provided will be treated confidentially. You are at liberty to withdraw from the study at any point in time and will not be penalized in any form for doing so. Thanks for agreeing to be part of this study.

**Socio-demographics**

1. Age/ Date of birth:
2. Gender (Male/ Female/ Other):
3. Profession:
4. Working Years in the Profession:
5. Educational Qualification:
6. Professional Rank:
7. Marital Status:
8. Number of children/dependents who seek healthcare at this facility:
9. Current College/Faculty/Department/Unit within KNUST:
10. Working Years in Current College/Faculty/Department/Unit within KNUST:

**Preliminary Questions**

1. How long have you been working in this hospital?
2. What are your experiences in providing care for OPD patients?

Prompts: How does the current OPD system influence your delivery of care and interactions with patients?- Time spent with patients, workload, organization of activities, care delivery, etc

3. What are your views/ opinions about the current OPD system whereby patients and/or their families do not have a specific doctor assigned to them (and are seen by any doctor on duty)?

Prompts: Advantages and disadvantages/challenges of the current system

4. What are your views on patient satisfaction with the current mode of care delivery based on your experiences and observations?
5. As a healthcare professional, to what extent are you satisfied with the current OPD system whereby patients and/or their families do not have a specific doctor assigned to them (and are seen by any doctor on duty)?

6. In your opinion, what are some of the measures that can be put in place to improve the care of patients at the OPD of this hospital?

**Brief Information about the Family Practice Model of Healthcare**

Family practice model of healthcare is a system whereby a patient and their family members are seen by one doctor. This system has been shown to be cost-effective and efficient in providing quality and continuous healthcare for patients and their families.

As part of efforts to improve the efficiency and effectiveness of OPD services and healthcare in general at this hospital, we want to introduce the family practice model of care and would be grateful to have your input on your perceptions and expectation of this model.

**Main study questions**

1. What is your opinion on the introduction of the family practice model (FPM) of care in the OPD system of this hospital?
2. If this system is introduced, would you prefer to be allocated to patients by the hospital or you would want the patients to choose their family doctors? Please provide a reason for your choice.
3. What factors should be considered in the selection of a family practice?

Prompts: gender, age, attributes/character, competencies, etc.

4. In your view, how many patients/families should be allocated to one doctor in this model? Please provide a reason for your answer.
5. What advantages do you perceive in having a family practice model (FPM) of OPD care compared to the current system whereby you and your family do not have a specific doctor assigned to them (and are seen by any doctor on duty)?

Prompts: Advantages of FPM to patients, families and healthcare professionals.

6. What challenges or drawbacks do you anticipate in the proposed family practice model of OPD care?

Prompts: Delays in accessing care, prolonged waiting times, insufficient provider competencies in caring for all body systems and all categories of family members, etc.

7. In what ways can the above-mentioned challenges be addressed?
8. Please do you have any other comments that were not addressed by the questions?

**Thanks for participating!**
