# Supplementary material for: Exploring patient and provider perspectives on outpatient care and the family practice model at a university hospital in Ghana
Source: PLoS One. 2026 Sep 9;21(9):e0327277. doi: 10.1371/journal.pone.0327277 (PMC13557406; doi:10.1371/journal.pone.0327277)
Supplement: Supplementary file 5 [file pone.0327277.s004.docx]

**Exploring patient and provider perspectives on outpatient care and the family practice model at a university hospital in Ghana**

Patients’ and providers’ perspectives on outpatient care in Ghana are important and well-placed to improve clinical practice and patient satisfaction.

**General Comments for Authors**

1. It is confusing to combine the quotes for the two groups of participants without referencing whether they were either healthcare providers or recipients under the same themes and sub-themes. You could adequately do so by referencing participants' quotes and describing the findings in a complementary way, or by analyzing the data for each group separately to ensure homogeneity and combining them during the discussion.

**Specific Comments**

| Number | Comment/suggestions | Page |
| --- | --- | --- |
| 1. | Expert purposive sampling could have been used for healthcare providers based on their work or where they work.  Healthcare recipients could have been recruited through convenience sampling to reach several university staff who were accessing healthcare at that time. Non-university staff could be excluded. | 171 – 176  177 - 186 |
| 2 | Did you get a translator for the Twi transcription? | 205 |
| 3 | Why did the data collection take so long? | 203 |
| 4 | Were the pretests conducted among healthcare providers or recipients? How many of each group of participants were pretested before the study | 217 |
| 5 | Your reason for using a hybrid approach to your thematic analysis while using a model is not clear | 233 |
| 6 | I am not sure of how you are analyzing your work. I initially thought you used the main constructs of the Consolidated Framework for Implementation research as your main themes (deductive), and the sub-themes were aligned with the study's constructs (inductive). However, I cannot find any construct of the model you have chosen; all I see are the inductive themes you have generated. The themes’ connections to the framework are not clear. | 320 - 329 |
| 7 | None of the participants' quotes was referenced. | 355 … |
| 8 | Be consistent with the spacing between the numbers and percentages | 309 |
| 9 | The table did not specify the population whose themes and subthemes were generated | 329 |
| 10 | When was data saturation achieved for each of the two groups of participants? |  |
