# Supplementary material for: Exploring patient and provider perspectives on outpatient care and the family practice model at a university hospital in Ghana
Source: PLoS One. 2026 Sep 9;21(9):e0327277. doi: 10.1371/journal.pone.0327277 (PMC13557406; doi:10.1371/journal.pone.0327277)
Supplement: Peer review history

# Peer review: Decision Letter 0

**DOI:** 10.1371/journal.pone.0327277.r001

**Authors:** Bismark Dwumfour-Asare

7 Oct 2025

Dear Dr. Kusi-Amponsah Diji,

Please submit your revised manuscript by Nov 21 2025 11:59PM. If you will need more time than this to complete your revisions, please reply to this message or contact the journal office at plosone@plos.org. When you're ready to submit your revision, log on to https://www.editorialmanager.com/pone/ and select the 'Submissions Needing Revision' folder to locate your manuscript file.

- A rebuttal letter that responds to each point raised by the academic editor and reviewer(s). You should upload this letter as a separate file labeled 'Response to Reviewers'.
- A marked-up copy of your manuscript that highlights changes made to the original version. You should upload this as a separate file labeled 'Revised Manuscript with Track Changes'.
- An unmarked version of your revised paper without tracked changes. You should upload this as a separate file labeled 'Manuscript'.

If you would like to make changes to your financial disclosure, please include your updated statement in your cover letter. Guidelines for resubmitting your figure files are available below the reviewer comments at the end of this letter.

If applicable, we recommend that you deposit your laboratory protocols in protocols.io to enhance the reproducibility of your results. Protocols.io assigns your protocol its own identifier (DOI) so that it can be cited independently in the future. For instructions see: https://journals.plos.org/plosone/s/submission-guidelines#loc-laboratory-protocols. Additionally, PLOS ONE offers an option for publishing peer-reviewed Lab Protocol articles, which describe protocols hosted on protocols.io. Read more information on sharing protocols at https://plos.org/protocols?utm_medium=editorial-email&utm_source=authorletters&utm_campaign=protocols.

We look forward to receiving your revised manuscript.

Kind regards,

Bismark Dwumfour-Asare, MSc, PhD

Academic Editor

PLOS ONE

Journal Requirements:

When submitting your revision, we need you to address these additional requirements.

1.Please ensure that your manuscript meets PLOS ONE's style requirements, including those for file naming. The PLOS ONE style templates can be found at

https://journals.plos.org/plosone/s/file?id=wjVg/PLOSOne_formatting_sample_main_body.pdf and

https://journals.plos.org/plosone/s/file?id=ba62/PLOSOne_formatting_sample_title_authors_affiliations.pdf

2. We note that your Data Availability Statement is currently as follows: All relevant data are within the manuscript and in Supporting Information files.

Please confirm at this time whether or not your submission contains all raw data required to replicate the results of your study. Authors must share the “minimal data set” for their submission. PLOS defines the minimal data set to consist of the data required to replicate all study findings reported in the article, as well as related metadata and methods (https://journals.plos.org/plosone/s/data-availability#loc-minimal-data-set-definition).

For example, authors should submit the following data:

- The values behind the means, standard deviations and other measures reported;

- The values used to build graphs;

- The points extracted from images for analysis.

Authors do not need to submit their entire data set if only a portion of the data was used in the reported study.

If your submission does not contain these data, please either upload them as Supporting Information files or deposit them to a stable, public repository and provide us with the relevant URLs, DOIs, or accession numbers. For a list of recommended repositories, please see https://journals.plos.org/plosone/s/recommended-repositories.

If there are ethical or legal restrictions on sharing a de-identified data set, please explain them in detail (e.g., data contain potentially sensitive information, data are owned by a third-party organization, etc.) and who has imposed them (e.g., an ethics committee). Please also provide contact information for a data access committee, ethics committee, or other institutional body to which data requests may be sent. If data are owned by a third party, please indicate how others may request data access.

3. We note that this data set consists of interview transcripts. Can you please confirm that all participants gave consent for interview transcript to be published?

If they DID provide consent for these transcripts to be published, please also confirm that the transcripts do not contain any potentially identifying information (or let us know if the participants consented to having their personal details published and made publicly available). We consider the following details to be identifying information:

- Names, nicknames, and initials

- Age more specific than round numbers

- GPS coordinates, physical addresses, IP addresses, email addresses

- Information in small sample sizes (e.g. 40 students from X class in X year at X university)

- Specific dates (e.g. visit dates, interview dates)

- ID numbers

Or, if the participants DID NOT provide consent for these transcripts to be published:

- Provide a de-identified version of the data or excerpts of interview responses

- Provide information regarding how these transcripts can be accessed by researchers who meet the criteria for access to confidential data, including:

a) the grounds for restriction

b) the name of the ethics committee, Institutional Review Board, or third-party organization that is imposing sharing restrictions on the data

c) a non-author, institutional point of contact that is able to field data access queries, in the interest of maintaining long-term data accessibility.

d) Any relevant data set names, URLs, DOIs, etc. that an independent researcher would need in order to request your minimal data set.

For further information on sharing data that contains sensitive participant information, please see: https://journals.plos.org/plosone/s/data-availability#loc-human-research-participant-data-and-other-sensitive-data

If there are ethical, legal, or third-party restrictions upon your dataset, you must provide all of the following details (https://journals.plos.org/plosone/s/data-availability#loc-acceptable-data-access-restrictions):

1. A complete description of the dataset

2. The nature of the restrictions upon the data (ethical, legal, or owned by a third party) and the reasoning behind them

3. The full name of the body imposing the restrictions upon your dataset (ethics committee, institution, data access committee, etc)

4. If the data are owned by a third party, confirmation of whether the authors received any special privileges in accessing the data that other researchers would not have

5. Direct, non-author contact information (preferably email) for the body imposing the restrictions upon the data, to which data access requests can be sent

4. If the reviewer comments include a recommendation to cite specific previously published works, please review and evaluate these publications to determine whether they are relevant and should be cited. There is no requirement to cite these works unless the editor has indicated otherwise.

Additional Editor Comments:

The topic is interesting and important to the health sector at large to learn from existing practice and experience. The study could be very useful to inform policy.

However, there are fundamental issues with the methodology and authors will have to address them.

Some of the issues are as follows:

- Study adopted a limited number of study participants for a purposive sampling without any justification nor adequate criteria. It is not convincing that the findings from such a limited study can support the conclusion and recommendations made for policy and practice.

Inadequate justification for choosing such a study design to assess a whole OPD system of a unique hospital facility serving a university community and other 4-5 general communities. Instead of choosing a more representative study design (including participants) to support a generalization.

Unfortunately, the criteria for selection of participants is unclear - especially patients, it’s not clear why only staff of the university were chosen. What about the larger general communities that authors claim are served by the hospital?

it's emphasising the limitation that the university staff may be receiving different level of service, may be preferentially different from the general community members. What was the interest or the purpose of the study, is it to satisfy only staff patients? meanwhile we know that staff OPD is clearly different from the general public. Personally, I have visited the facility several times and I have a course to believe that I am treated differently at the OPD compared to others who are staff of the university.

A cross-sectional study involving questionnaire administration would have helped improve data collection? Better still, with the same qualitative approach, couldn’t the use of focus groups with some community patients/members have helped to bring their perspective to bear?

Meanwhile, no evidence is presented that participant selection achieved any theoretical saturation with respect to the emerging themes or findings expected from a qualitative study.

Again, no limitations enumerated openly for readers to appreciate that authors have acknowledged those that were beyond their control - obviously there are several of such with this kind of study design that the authors failed to indicate them.

Also, these comments should be considered for revision:

Abstract:

o Keywords too loaded, should consider standard keywords in the discipline using less of 3 words phrases.

o background of abstract too lengthy, it should be more focused and concise.

o avoid use of acronyms in abstract FPM for first time use

[Note: HTML markup is below. Please do not edit.]

Reviewers' comments:

Reviewer's Responses to Questions

**Comments to the Author**

1. Is the manuscript technically sound, and do the data support the conclusions?

Reviewer #1: Yes

Reviewer #2: Yes

**********

2. Has the statistical analysis been performed appropriately and rigorously?

Reviewer #1: Yes

Reviewer #2: N/A

**********

3. Have the authors made all data underlying the findings in their manuscript fully available?

The PLOS Data policy

Reviewer #1: Yes

Reviewer #2: Yes

**********

4. Is the manuscript presented in an intelligible fashion and written in standard English?

Reviewer #1: Yes

Reviewer #2: Yes

**********

Reviewer #1: 1. The manuscript is technically sound scientifically with data supporting the conclusion.

2. Statistical Qualitative data analysis has been performed appropriately.

3. Authors made all data underlying the findings in their manuscript fully available.

4. The manuscript has been presented in an intelligible fashion and written in standard English. However the authors should consider reviewing some grammatical errors. Foreample, the first paragraph in the introduction section with reads "Primary healthcare is widely recognized as the cornerstone of effective health systems, as it substantially improves access to essential services, strengthens disease prevention, and supports coordinated care over" seems to be incomplete and might confuse the readers.

Reviewer #2: This is a well-designed and highly relevant study that adds crucial context-specific evidence regarding primary care strengthening in Ghana. The use of the CFIR framework is a significant strength, providing structure to the findings that will be valuable for policymakers and implementation scientists.

Major Comments

1. Clarify Study Duration (Critical): Please confirm and correct the discrepancy between the study duration stated in the Abstract (April to May 2024) and the Methods section (September 2023 to May 2024). This is a technical error that must be resolved.

2. Integration of CFIR in Discussion: While the CFIR is effectively introduced in the Methods, the Discussion section should be structured to explicitly link the identified themes and sub-themes back to the relevant CFIR domains (e.g., Inner Setting, Characteristics of Individuals, Intervention Characteristics). For instance:

-The "Therapeutic Disconnection" theme relates directly to a weak Inner Setting (poor documentation, rushed consultations).

- The "Beneath the Optimism" sub-theme clearly relates to Outer Setting (patient needs for flexibility) and Inner Setting (workload, resource availability).

- Strengthening this discussion will maximize the manuscript's contribution to implementation science.

Minor Comments

1. Sampling Justification: The authors note that care recipients were university staff. While purposive sampling is justified, briefly explain why this specific group of care recipients (university staff) was chosen over a broader sample from the community or general hospital users. Does this reflect a targeted initial implementation phase or a specific population served by the hospital?

2. Illustrative Quotes: The manuscript includes excellent quotes (e.g., "You don’t have time to breathe. You just keep going..." ). Please ensure all main sub-themes (e.g., "Care on the clock," "Fragmented care," "Care without comfort," "Healing through continuity") are supported by direct, clearly cited quotes within the results section for maximum impact and transparency.

3. Future Research/Pilot Testing: The conclusion mentions the need for pilot testing. It would be beneficial to briefly suggest specific, measurable outcomes that future pilot studies could use to assess FPM feasibility and impact (e.g., reduced hospitalization rates, improved chronic disease control, change in trust scores).

**********

what does this mean?). If published, this will include your full peer review and any attached files.

If you choose “no”, your identity will remain anonymous but your review may still be made public.

**Do you want your identity to be public for this peer review?**  For information about this choice, including consent withdrawal, please see our Privacy Policy

Reviewer #1: **Yes:**  Oscar Mahinyila

Reviewer #2: **Yes:**  Mohammad J. Alhawajreh

**********

[NOTE: If reviewer comments were submitted as an attachment file, they will be attached to this email and accessible via the submission site. Please log into your account, locate the manuscript record, and check for the action link "View Attachments". If this link does not appear, there are no attachment files.]

While revising your submission, please upload your figure files to the Preflight Analysis and Conversion Engine (PACE) digital diagnostic tool, https://pacev2.apexcovantage.com/. PACE helps ensure that figures meet PLOS requirements. To use PACE, you must first register as a user. Registration is free. Then, login and navigate to the UPLOAD tab, where you will find detailed instructions on how to use the tool. If you encounter any issues or have any questions when using PACE, please email PLOS at figures@plos.org. Please note that Supporting Information files do not need this step.

# Author comment: Author response to Decision Letter 1

**DOI:** 10.1371/journal.pone.0327277.r002

20 Apr 2026

RESPONSE TO REVIEWERS

First reviewer

Comment

1.Please ensure that your manuscript meets PLOS ONE's style requirements, including those for file naming.

Response

We appreciate the reminder regarding PLOS ONE’s formatting and style requirements. The entire manuscript has been reviewed and reformatted to comply with the PLOS ONE style guidelines. Specifically:

The main text, tables, and references have been formatted according to the PLOS ONE sample templates provided.

The title page, including author names, affiliations, and corresponding author details, now conforms to the required PLOS ONE format.

File names have been revised to meet PLOS ONE naming conventions.

Comment

2. We note that your Data Availability Statement is currently as follows: All relevant data are within the manuscript and in Supporting Information files.

Please confirm at this time whether or not your submission contains all raw data required to replicate the results of your study. Authors must share the “minimal data set” for their submission. PLOS defines the minimal data set to consist of the data required to replicate all study findings reported in the article, as well as related metadata and methods (https://journals.plos.org/plosone/s/data-availability#loc-minimal-data-set-definition).

For example, authors should submit the following data:

- The values behind the means, standard deviations and other measures reported;

- The values used to build graphs;

- The points extracted from images for analysis.

Authors do not need to submit their entire data set if only a portion of the data was used in the reported study.

If your submission does not contain these data, please either upload them as Supporting Information files or deposit them to a stable, public repository and provide us with the relevant URLs, DOIs, or accession numbers. For a list of recommended repositories, please see https://journals.plos.org/plosone/s/recommended-repositories.

If there are ethical or legal restrictions on sharing a de-identified data set, please explain them in detail (e.g., data contain potentially sensitive information, data are owned by a third-party organization, etc.) and who has imposed them (e.g., an ethics committee). Please also provide contact information for a data access committee, ethics committee, or other institutional body to which data requests may be sent. If data are owned by a third party, please indicate how others may request data access.

Response

Thank you very much. We confirm that all relevant data required to replicate the findings of this qualitative study are contained within the manuscript and the accompanying Supporting Information files (S1–S3 Appendices).

Comment

3. We note that this data set consists of interview transcripts. Can you please confirm that all participants gave consent for interview transcript to be published?

If they DID provide consent for these transcripts to be published, please also confirm that the transcripts do not contain any potentially identifying information (or let us know if the participants consented to having their personal details published and made publicly available). We consider the following details to be identifying information:

- Names, nicknames, and initials

- Age more specific than round numbers

- GPS coordinates, physical addresses, IP addresses, email addresses

- Information in small sample sizes (e.g. 40 students from X class in X year at X university)

- Specific dates (e.g. visit dates, interview dates)

- ID numbers

Response

Thank you. Please we confirm that all participants provided written informed consent prior to their participation, including consent for their anonymized interview transcripts to be used and published for research purposes.

The transcripts have been de-identified to maintain confidentiality and privacy. They do not contain any potentially identifying information such as names, nicknames, initials, specific ages, physical or email addresses, GPS coordinates, visit or interview dates, or ID numbers.

Additional comments:

Comment

The topic is interesting and important to the health sector at large to learn from existing practice and experience. The study could be very useful to inform policy.

However, there are fundamental issues with the methodology and authors will have to address them.

Some of the issues are as follows:

Response

Please we appreciate your observations on the methodological aspects of our study. We have addressed these concerns comprehensively in the revised manuscript.

Comment

1. Study adopted a limited number of study participants for a purposive sampling without any justification nor adequate criteria. It is not convincing that the findings from such a limited study can support the conclusion and recommendations made for policy and practice.

Response

Thank you for this comment. We wish to clarify that the participants selected were people who constituted the hospital’s core and had direct experience with the OPD system whose opinions would be useful in piloting the FPM before extending it to surrounding communities (see: participant recruitment, page 8-10).

Comment

2. Inadequate justification for choosing such a study design to assess a whole OPD system of a unique hospital facility serving a university community and other 4-5 general communities. Instead of choosing a more representative study design (including participants) to support a generalization.

Response

Thank you. We wish to clarify that our main aim for choosing this study design was to capture the in-depth views and insights of key stakeholders for which the introduction of FPM would be piloted. (see study design section, page 7).

Comment

3.Unfortunately, the criteria for selection of participants is unclear - especially patients, it’s not clear why only staff of the university were chosen. What about the larger general communities that authors claim are served by the hospital?

Response

Thank you. Please our study focused on university staff (senior members, senior staffs and junior members) because they represent the hospital’s core and most consistent outpatient population, whose experiences typify the dominant service patterns at the University Hospital, KNUST. Given that the Family Practice Model (FPM) is being considered for initial implementation within the university hospital system, targeting this group allowed for a controlled exploration of feasibility and acceptability before potential extension to the wider surrounding communities (see Participant recruitment section, page 8-10).

Comment

it's emphasising the limitation that the university staff may be receiving different level of service, may be preferentially different from the general community members. What was the interest or the purpose of the study, is it to satisfy only staff patients? meanwhile we know that staff OPD is clearly different from the general public. Personally, I have visited the facility several times and I have a course to believe that I am treated differently at the OPD compared to others who are staff of the university.

Response

The purpose of this study was not to assess satisfaction among staff patients alone, but rather to explore the feasibility, acceptability, and contextual fit of the proposed Family Practice Model (FPM) within the University Hospital, KNUST. The decision to focus on university staff was intentional and based on two reasons. First, the FPM is intended for pilot implementation within the hospital’s core outpatient population primarily university staffs, before potential scale-up to the general public. Second, university staff represent a stable and accessible population with consistent service utilization patterns, making them appropriate for initial qualitative exploration of the model.

We agree that staff may experience care dynamics that differ from those of the general public, and this has been acknowledged as a limitation in the revised. Refer to Page 38, lines 779-791.

Comment

4.A cross-sectional study involving questionnaire administration would have helped improve data collection? Better still, with the same qualitative approach, couldn’t the use of focus groups with some community patients/members have helped to bring their perspective to bear?

Meanwhile, no evidence is presented that participant selection achieved any theoretical saturation with respect to the emerging themes or findings expected from a qualitative study.

Response

Thank you for this suggestion. However, the study’s qualitative design was deliberately chosen to explore, in depth, the nuanced experiences and expectations of key stakeholders for which the FPM introduction will be piloted on.

We acknowledge that focus group discussions could have enriched the dataset, individual in-depth interviews were prioritized to ensure participants could freely express potentially sensitive or critical views about existing care systems without peer influence or hierarchical pressure, particularly relevant in a university hospital setting where providers and recipients often share institutional affiliations.

Regarding theoretical saturation, data collection and analysis were conducted concurrently, and recruitment continued until no new themes or insights emerged. This point of thematic saturation was reached after the 25th interview, consistent with guidance for qualitative inquiry of this nature (Wultich et al., 2024). Refer to the methods section on page 11, lines 231-233).

Comment

5.Again, no limitations enumerated openly for readers to appreciate that authors have acknowledged those that were beyond their control - obviously there are several of such with this kind of study design that the authors failed to indicate them.

Response

We appreciate this observation and agree that stating the study’s limitations strengthens the transparency and interpretability of our findings. A dedicated paragraph has been added to the Discussion section to acknowledge methodological and contextual limitations, such as restricted participant scope, potential social desirability bias, and limited generalizability inherent to qualitative inquiry. Refer to the Limitations section on page 38, line 779-791.

Comment

6. Also, these comments should be considered for revision:

Abstract:

o Keywords too loaded, should consider standard keywords in the discipline using less of 3 words phrases.

o background of abstract too lengthy, it should be more focused and concise.

o avoid use of acronyms in abstract FPM for first time use

Response

Thank you for your feedback. Please we have revised the abstract to make the background more concise and focused on the study purpose, avoided the use of acronyms on first mention, and replaced overly specific multi-word phrases in the keywords with standardized, discipline-appropriate terms to enhance discoverability. See Abstract section on page 2).

Comment

4. The manuscript has been presented in an intelligible fashion and written in standard English. However, the authors should consider reviewing some grammatical errors. For example, the first paragraph in the introduction section with reads "Primary healthcare is widely recognized as the cornerstone of effective health systems, as it substantially improves access to essential services, strengthens disease prevention, and supports coordinated care over" seems to be incomplete and might confuse the readers.

Response

Thank you. The incomplete sentence in the Introduction has been corrected to improve clarity and flow. The revised sentence now reads:

“Primary healthcare is widely recognized as the cornerstone of effective health systems, as it enhances access to essential services, strengthens disease prevention, and supports coordinated care throughout a person’s life course”

Additionally, the entire manuscript has been carefully proofread to address minor grammatical and stylistic issues for enhanced readability (see introduction, page3).

Second Reviewer

Comment

1. Clarify Study Duration (Critical): Please confirm and correct the discrepancy between the study duration stated in the Abstract (April to May 2024) and the Methods section (September 2023 to May 2024). This is a technical error that must be resolved.

Response

Please, we have reviewed the manuscript and confirm that the correct study duration is September 2023 to May 2024. This period reflects the entire timeline of data collection and analysis.

(see: data collection and analysis; refer to page 10, lines 203-205).

Comment

2. Integration of CFIR in Discussion: While the CFIR is effectively introduced in the Methods, the Discussion section should be structured to explicitly link the identified themes and sub-themes back to the relevant CFIR domains (e.g., Inner Setting, Characteristics of Individuals, Intervention Characteristics). For instance:

-The "Therapeutic Disconnection" theme relates directly to a weak Inner Setting (poor documentation, rushed consultations).

- The "Beneath the Optimism" sub-theme clearly relates to Outer Setting (patient needs for flexibility) and Inner Setting (workload, resource availability).

- Strengthening this discussion will maximize the manuscript's contribution to implementation science.

Response

Please we have revised the Discussion section to clearly map each major theme and sub-theme to the relevant CFIR domains to enhance conceptual clarity and strengthen the study’s contribution to implementation science. Refer to the discussion section on page 34-38.

Comment

1. Sampling Justification: The authors note that care recipients were university staff. While purposive sampling is justified, briefly explain why this specific group of care recipients (university staff) was chosen over a broader sample from the community or general hospital users. Does this reflect a targeted initial implementation phase or a specific population served by the hospital?

Response

Please the selection of university staff as care recipients was intentional and based on the hospital’s primary service population. The proposed FPM is expected to be piloted internally for university staffs before broader community implementation. This purposive sampling thus ensured relevance to the hospital’s operational context and the study’s exploratory objectives.

Comment

2. Illustrative Quotes: The manuscript includes excellent quotes (e.g., "You don’t have time to breathe. You just keep going...”). Please ensure all main sub-themes (e.g., "Care on the clock," "Fragmented care," "Care without comfort," "Healing through continuity") are supported by direct, clearly cited quotes within the results section for maximum impact and transparency.

Response

Thank you for this kind suggestion. However, we have ensured that each main sub-theme, including “Care on the clock,” “Fragmented care,” “Care without comfort,” and “Healing through continuity” is supported by direct, clearly cited illustrative quotes from participants. Refer to Pages 19-33.

Comment

3. Future Research/Pilot Testing: The conclusion mentions the need for pilot testing. It would be beneficial to briefly suggest specific, measurable outcomes that future pilot studies could use to assess FPM feasibility and impact (e.g., reduced hospitalization rates, improved chronic disease control, change in trust scores).

Response

Thank you. We have revised the conclusion to specify measurable outcomes that future pilot studies could employ to assess the feasibility and impact of the Family Physician Model (FPM). These include indicators such as reduced hospitalization and readmission rates, improved chronic disease control (e.g., blood pressure and glucose monitoring outcomes), enhanced patient satisfaction and trust scores, and provider workload balance. Refer to the conclusion section on page 39, lines 801 to 804.

Attachment Submitted filename: Response to Review Comments_April 2026.pdf

# Peer review: Decision Letter 1

**DOI:** 10.1371/journal.pone.0327277.r003

**Authors:** Immaculate Sabelile Tenza

12 May 2026

Dear Dr. Kusi-Amponsah Diji,

Please submit your revised manuscript by Jun 26 2026 11:59PM. If you will need more time than this to complete your revisions, please reply to this message or contact the journal office at plosone@plos.org. When you're ready to submit your revision, log on to https://www.editorialmanager.com/pone/ and select the 'Submissions Needing Revision' folder to locate your manuscript file.

- A letter that responds to each point raised by the academic editor and reviewer(s). You should upload this letter as a separate file labeled 'Response to Reviewers'.
- A marked-up copy of your manuscript that highlights changes made to the original version. You should upload this as a separate file labeled 'Revised Manuscript with Track Changes'.
- An unmarked version of your revised paper without tracked changes. You should upload this as a separate file labeled 'Manuscript'.

If you would like to make changes to your financial disclosure, please include your updated statement in your cover letter. Guidelines for resubmitting your figure files are available below the reviewer comments at the end of this letter.

If applicable, we recommend that you deposit your laboratory protocols in protocols.io to enhance the reproducibility of your results. Protocols.io assigns your protocol its own identifier (DOI) so that it can be cited independently in the future. For instructions see: https://journals.plos.org/plosone/s/submission-guidelines#loc-laboratory-protocols. Additionally, PLOS ONE offers an option for publishing peer-reviewed Lab Protocol articles, which describe protocols hosted on protocols.io. Read more information on sharing protocols at https://plos.org/protocols?utm_medium=editorial-email&utm_source=authorletters&utm_campaign=protocols.

As the corresponding author, your ORCID iD is verified in the submission system and will appear in the published article. PLOS supports the use of ORCID, and we encourage all coauthors to register for an ORCID iD and use it as well. Please encourage your coauthors to verify their ORCID iD within the submission system before final acceptance, as unverified ORCID iDs will not appear in the published article. *Only*  the individual author can complete the verification step; PLOS staff *cannot*  verify ORCID iDs on behalf of authors.

We look forward to receiving your revised manuscript.

Kind regards,

Immaculate Sabelile Tenza, PhD

Academic Editor

PLOS One

Journal Requirements:

If the reviewer comments include a recommendation to cite specific previously published works, please review and evaluate these publications to determine whether they are relevant and should be cited. There is no requirement to cite these works unless the editor has indicated otherwise.

Additional Editor Comments (if provided):

Dear Authors, kindly address the comments from the reviewers

[Note: HTML markup is below. Please do not edit.]

Reviewers' comments:

Reviewer's Responses to Questions

**Comments to the Author**

Reviewer #2: All comments have been addressed

Reviewer #3: (No Response)

**********

2. Is the manuscript technically sound, and do the data support the conclusions?

Reviewer #2: Yes

Reviewer #3: No

**********

3. Has the statistical analysis been performed appropriately and rigorously?

Reviewer #2: N/A

Reviewer #3: N/A

**********

4. Have the authors made all data underlying the findings in their manuscript fully available?

The PLOS Data policy

Reviewer #2: Yes

Reviewer #3: No

**********

5. Is the manuscript presented in an intelligible fashion and written in standard English?

Reviewer #2: Yes

Reviewer #3: No

**********

Reviewer #2: (No Response)

Reviewer #3: (No Response)

**********

what does this mean?). If published, this will include your full peer review and any attached files.

If you choose “no”, your identity will remain anonymous but your review may still be made public.

**Do you want your identity to be public for this peer review?**  For information about this choice, including consent withdrawal, please see our Privacy Policy

Reviewer #2: **Yes:**  Dr. Mohammad J. Alhawajreh

Reviewer #3: No

**********

[NOTE: If reviewer comments were submitted as an attachment file, they will be attached to this email and accessible via the submission site. Please log into your account, locate the manuscript record, and check for the action link "View Attachments". If this link does not appear, there are no attachment files.]

To ensure your figures meet our technical requirements, please review our figure guidelines: https://journals.plos.org/plosone/s/figures

You may also use PLOS’s free figure tool, NAAS, to help you prepare publication quality figures: https://journals.plos.org/plosone/s/figures#loc-tools-for-figure-preparation.

NAAS will assess whether your figures meet our technical requirements by comparing each figure against our figure specifications.

Attachment Submitted filename: perspectives on outpatient care12052026.TDA.docx

# Author comment: Author response to Decision Letter 2

**DOI:** 10.1371/journal.pone.0327277.r004

5 Aug 2026

Exploring patient and provider perspectives on outpatient care and the family practice model at a university hospital in Ghana

Response to Reviewers’ Comments

We thank the reviewer(s) for the constructive comments and suggestions. We have revised the manuscript accordingly. Each reviewer comment is reproduced below, followed immediately by our response and a statement of the corresponding change made in the revised manuscript, including the relevant page or line reference where available.

Comment: Expert purposive sampling could have been used for healthcare providers based on their work or where they work.

Response: Thank you for the comment. However, we wish to clarify that we used purposive sampling with a maximum variation strategy to recruit healthcare providers, specifically targeting medical officers and physician assistants with at least one year of experience in the hospital OPD. This ensured inclusion of information-rich participants with direct experience of the system under study. While expert purposive sampling could also have been appropriate, our approach was chosen to capture a broader range of provider perspectives across roles and units rather than limiting selection to a narrower expert subgroup. Refer to Lines 171-176.

Comment: Healthcare recipients could have been recruited through convenience sampling to reach several university staff who were accessing healthcare at that time. Non-university staff could be excluded.

Response: That is very thoughtful. However, healthcare recipients were purposively recruited among university staff who had accessed OPD services within the past year. This was intentional to ensure participants had relevant, recent experience with the system under study. We opted against convenience sampling to avoid capturing only those present at the health facility at the time of data collection, which could have introduced selection bias and limited variation in experiences. Restricting inclusion to university staff was also deliberate, as they represent a stable and consistent user group of the hospital’s OPD services, which was important for the objectives of this study.

Comment: Did you get a translator for the Twi transcription?

Response: Thanks for the comment. Clarification on the process has been provided in the revised manuscript as follows:

Interviews conducted in Twi were transcribed and translated into English by bilingual members of the research team who were fluent in both languages. The accuracy of the translations was validated by a Twi language expert, and all transcripts were cross-checked against the original audio recordings to ensure fidelity and accuracy. Refer to Lines 206-209.

Comment: Why did the data collection take so long?

Response: Thank you for this observation. We wish to clarify that the data collection period, from September 2023 to May 2024, reflected the practical and methodological demands of the study rather than delays in the research process. The study involved two stakeholder groups: healthcare providers and university staff who had accessed OPD services. Many participants had demanding and variable clinical, administrative, and academic schedules; therefore, interviews had to be arranged at times and locations convenient to them to minimise disruption to their duties.

In addition, data collection was conducted concurrently with transcription, translation where necessary, and preliminary analysis. This iterative process allowed the research team to refine interview probes, compare emerging insights across participant groups, and continue recruitment until thematic saturation was achieved. The extended period was therefore necessary to ensure adequate participant diversity, depth of inquiry, reflexivity, and methodological rigour. Refer to Lines 203, 233-234.

Comment: Were the pretests conducted among healthcare providers or recipients? How many of each group of participants were pretested before the study?

Response: Thank you for this important observation. The interview guides were pretested among both participant groups prior to the main study. Specifically, the pretest involved seven healthcare providers and seven healthcare recipients from a similar hospital setting.

Their feedback was used to improve the clarity, sequencing, relevance and flow of the questions as the overall focus of the guide remained unchanged. See Lines 220-223.

Comment: Your reason for using a hybrid approach to your thematic analysis while using a model is not clear.

Response: Thank you for this comment. We adopted a hybrid thematic analysis approach to ensure that the analysis was guided by the conceptual model without being constrained by it. The model provided a deductive structure for examining issues central to the study, while inductive coding allowed unanticipated and context-specific insights to emerge from participants’ narratives. This approach enhanced analytical rigour by ensuring that the findings were both theoretically informed and grounded in the empirical data. Refer to Lines 240-245.

Comment: I am not sure of how you are analyzing your work. I initially thought you used the main constructs of the Consolidated Framework for Implementation Research as your main themes (deductive), and the sub-themes were aligned with the study's constructs (inductive). However, I cannot find any construct of the model you have chosen; all I see are the inductive themes you have generated. The themes’ connections to the framework are not clear.

Response: Thank you. However, we wish to clarify that our analysis was guided by a hybrid approach rather than using CFIR constructs as preset themes. CFIR informed data collection, coding, and interpretation, but themes were derived inductively from participants’ accounts to preserve depth and context. To address the concern, we have strengthened the Results section by explicitly linking the themes to CFIR constructs. For example, Theme 1 to the Inner Setting (and relevant Outer Setting influences) and Theme 2 to Intervention Characteristics, Inner Setting, and Process domains, making the alignment with CFIR more transparent. Refer to Lines 339, 351-357, 365-367, 397-402, 458-462, 525-532, 542-544, 605-608, and 669-672.

Comment: None of the participants' quotes was referenced.

Response: Thank you for this observation. We have revised all quotations in the results section to clearly indicate the source of each statement, specifying whether it was provided by a healthcare provider or a healthcare recipient. Refer to Lines 373-726.

Comment: Be consistent with the spacing between the numbers and percentages.

Response: Thank you. We have reviewed the manuscript and corrected all instances to ensure consistent formatting of numbers and percentages throughout the text. See Pages 1-47.

Comment: The table did not specify the population whose themes and subthemes were generated.

Response: Thank you. We have revised Table 3 to clearly indicate that the themes and sub-themes were derived from both healthcare providers and healthcare recipients, who were university staff participating in the study. Refer to Line 339.

Comment: When was data saturation achieved for each of the two groups of participants?

Response: Thank you for this comment. Data saturation was reached at different points for the two participant groups. For healthcare providers, saturation was achieved after the eighth interview. For healthcare recipients, saturation was achieved after the 17th interview. However, interviews continued up to nine providers and 19 recipients to ensure full coverage and to confirm that no new themes or perspectives were emerging across either group. Refer to Lines 194-200.

Attachment Submitted filename: Response to Reviewers_25.06.26.pdf

# Editor report: Decision Letter 2

**DOI:** 10.1371/journal.pone.0327277.r005

**Authors:** Immaculate Sabelile Tenza

19 Aug 2026

Exploring patient and provider perspectives on outpatient care and the family practice model at a university hospital in Ghana

PONE-D-25-27394R2

Dear Dr. Kusi-Amponsah Diji,

We’re pleased to inform you that your manuscript has been judged scientifically suitable for publication and will be formally accepted for publication once it meets all outstanding technical requirements.

Within one week, you’ll receive an e-mail detailing the required amendments. When these have been addressed, you’ll receive a formal acceptance letter and your manuscript will be scheduled for publication.

An invoice will be generated when your article is formally accepted. Please note, if your institution has a publishing partnership with PLOS and your article meets the relevant criteria, all or part of your publication costs will be covered. Please make sure your user information is up-to-date by logging into Editorial Manager at Editorial Manager® and clicking the ‘Update My Information' link at the top of the page. For questions related to billing, please contact billing support.

If your institution or institutions have a press office, please notify them about your upcoming paper to help maximize its impact. If they’ll be preparing press materials, please inform our press team as soon as possible -- no later than 48 hours after receiving the formal acceptance. Your manuscript will remain under strict press embargo until 2 pm Eastern Time on the date of publication. For more information, please contact onepress@plos.org.

Kind regards,

Immaculate Sabelile Tenza, PhD

Academic Editor

PLOS One

Additional Editor Comments (optional):

Reviewers' comments:

# Editor report: Acceptance letter

**DOI:** 10.1371/journal.pone.0327277.r006

**Authors:** Immaculate Sabelile Tenza

PONE-D-25-27394R2

PLOS One

Dear Dr. Kusi-Amponsah Diji,

I'm pleased to inform you that your manuscript has been deemed suitable for publication in PLOS One. Congratulations! Your manuscript is now being handed over to our production team.

At this stage, our production department will prepare your paper for publication. This includes ensuring the following:

* All references, tables, and figures are properly cited

* All relevant supporting information is included in the manuscript submission,

* There are no issues that prevent the paper from being properly typeset

You will receive further instructions from the production team, including instructions on how to review your proof when it is ready. Please keep in mind that we are working through a large volume of accepted articles, so please give us a few days to review your paper and let you know the next and final steps.

Lastly, if your institution or institutions have a press office, please let them know about your upcoming paper now to help maximize its impact. If they'll be preparing press materials, please inform our press team within the next 48 hours. Your manuscript will remain under strict press embargo until 2 pm Eastern Time on the date of publication. For more information, please contact onepress@plos.org.

You will receive an invoice from PLOS for your publication fee after your manuscript has reached the completed accept phase. If you receive an email requesting payment before acceptance or for any other service, this may be a phishing scheme. Learn how to identify phishing emails and protect your accounts at https://explore.plos.org/phishing.

If we can help with anything else, please email us at customercare@plos.org.

Thank you for submitting your work to PLOS One and supporting open access.

Kind regards,

PLOS One Editorial Office Staff

on behalf of

Dr. Immaculate Sabelile Tenza

Academic Editor

PLOS One
